# Co-option of *Plasmodium falciparum* PP1 for egress from host erythrocytes

**DOI:** 10.1038/s41467-020-17306-1

**Published:** 2020-07-15

**Authors:** Aditya S. Paul, Alexandra Miliu, Joao A. Paulo, Jonathan M. Goldberg, Arianna M. Bonilla, Laurence Berry, Marie Seveno, Catherine Braun-Breton, Aziz L. Kosber, Brendan Elsworth, Jose S. N. Arriola, Maryse Lebrun, Steven P. Gygi, Mauld H. Lamarque, Manoj T. Duraisingh

**Affiliations:** 1 Department of Immunology and Infectious Diseases, Harvard T. H. Chan School of Public Health, Boston, 02115 MA USA; 2 Laboratory of Pathogen Host Interaction (LPHI), UMR5235, Centre National de la Recherche Scientifique (CNRS), Université de Montpellier, 34095 Montpellier, France; 3 Department of Cell Biology, Harvard Medical School, Boston, 02115 MA USA

**Keywords:** Parasite biology, Parasite development

## Abstract

Asexual proliferation of the *Plasmodium* parasites that cause malaria follows a developmental program that alternates non-canonical intraerythrocytic replication with dissemination to new host cells. We carried out a functional analysis of the *Plasmodium falciparum* homolog of Protein Phosphatase 1 (*Pf*PP1), a universally conserved cell cycle factor in eukaryotes, to investigate regulation of parasite proliferation. *Pf*PP1 is indeed required for efficient replication, but is absolutely essential for egress of parasites from host red blood cells. By phosphoproteomic and chemical-genetic analysis, we isolate two functional targets of *Pf*PP1 for egress: a HECT E3 protein-ubiquitin ligase; and GCα, a fusion protein composed of a guanylyl cyclase and a phospholipid transporter domain. We hypothesize that *Pf*PP1 regulates lipid sensing by GCα and find that phosphatidylcholine stimulates *Pf*PP1-dependent egress. *Pf*PP1 acts as a key regulator that integrates multiple cell-intrinsic pathways with external signals to direct parasite egress from host cells.

## Abstract (web summary)

*Plasmodium* protein phosphatase PP1 is essential for the asexual proliferation of malaria parasites. Here the authors show that PP1 regulates egress of parasites from host red blood cells, integrating parasite intrinsic pathways with environmental signals for release into the bloodstream.

## Introduction

Malaria parasites from the genus *Plasmodium* follow an unusual developmental program during infection of erythrocyte host cells, utilizing a non-canonical style of asexual proliferation known as schizogony to undergo multiple cycles of nuclear replication before a single cytokinesis event to form merozoites^1,2^. Merozoites infect new host cells to initiate new intraerythrocytic developmental cycles (IDC) and sustain proliferation, achieved only by egress from host cells for release into circulation. Protein phosphorylation in parasites is developmentally regulated in blood-stage growth^3^, and genetic studies show that roughly one-half of the *Plasmodium* protein kinase and protein phosphatase genes are essential for the IDC^4–8^.

Protein Phosphatase 1 (PP1) is a highly conserved and ubiquitous enzyme in eukaryotes that regulates mitotic exit and cytokinesis^9–11^. With functions also in non-cell cycle-related processes (reviewed in^12^), PP1 is a dominant contributor to total cellular phosphatase activity^13^. For the *Plasmodium falciparum* homolog of PP1 (*Pf*PP1)^14^, genetic evidence for likely essentiality^5,7^, high levels of expression^15,16^ (Supplementary Fig. 1a), and the identification of several binding proteins (e.g. Refs. ^17–21^), suggest multiple functions in asexual, blood-stage parasites.

We used a reverse genetic approach to carry out a functional analysis of *Pf*PP1 in asexual proliferation of *P. falciparum*. With conditional expression of the endogenously expressed enzyme, we establish essential functions for *Pf*PP1 during intraerythrocytic development and at egress. Phosphoproteomic and chemical-genetic analyses of essential *Pf*PP1 activity at egress indicate regulation of the functions of a HECT-family E3 ligase, and a putative phospholipid transporter fused to a guanylyl cyclase utilized for host cell rupture. Based on chemical-genetic and biochemical evidence, we propose that *Pf*PP1 regulates translocation of phosphatidylcholine across the phospholipid transporter and stimulates cGMP synthesis for egress.

## Results

### P*f*PP1 in parasite development and host cell egress

**Fig. 1:**
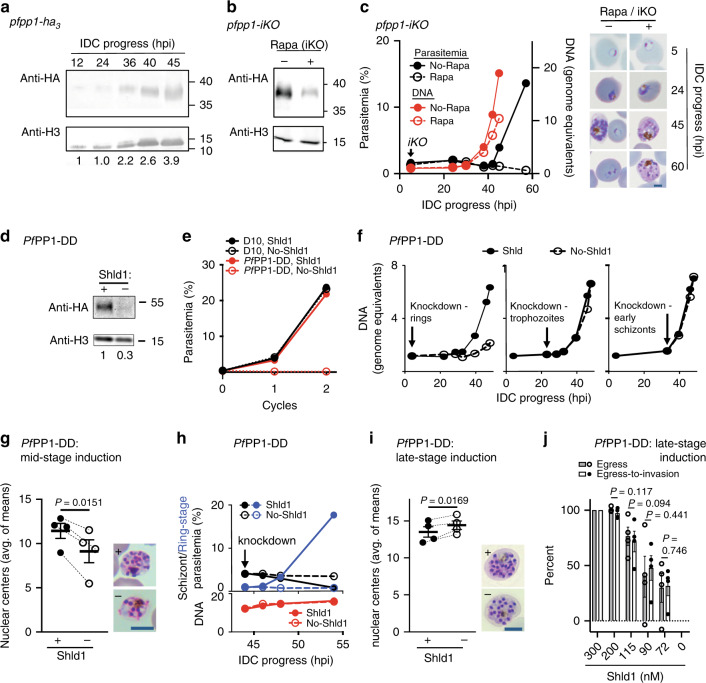
Isolating an essential P*f*PP1 function late in the intraerythrocytic developmental cycle. **a** *Pf*PP1-HA_3_ expression during intraerythrocytic development (hours post-invasion, hpi), assessed by immunoblot. Relative *Pf*PP1 levels (below lanes) are normalized to histone H3. Representative of 3 experiments. Molecular mass in kDa. **b** iKO of *pfpp1* was initiated at 5 hpi with rapamycin (Rapa) and protein levels were assessed by immunoblot at 30 hpi (sample processing control on separate gel). Representative of 2 experiments. Molecular mass in kDa. **c** Left: Parasitemia and DNA synthesis over the IDC following +/−Rapa-treatment at 5 hpi in *pfpp1-iKO* parasites, monitored by flow-cytometry. Mean of 3 technical replicates. Representative of 4 experiments. Right: Images of parasites along the IDC, following +/−Rapa-treatment at 5-hpi. Scale bar: 2 µm. **d** HA-tagged *Pf*PP1-DD protein from schizont-stage parasites (~48 hpi) grown +/−Shld1 for 6 h, assessed by immunoblot. Molecular mass in kDa. Representative of 1 experiment. **e** Proliferation of *Pf*PP1-DD and parental D10 parasites (wild type), +/−Shld1, monitored by flow-cytometry. Knockdown was induced in cycle-zero. Mean of 2 technical replicates. Representative of 2 experiments. **f** DNA replication in *Pf*PP1-DD parasites following knockdown at the indicated timepoints, monitored by flow-cytometry. Mean of 2 technical replicates. Representative of 4 experiments for ring-stage induction, 2 experiments for trophozoite-stage induction, and 2 experiments for early schizont-stage induction. **g** Left: Nuclear centers in terminally developed *Pf*PP1-DD parasites, assessed by light microscopy. Knockdown induced at 22-30 hpi. Mean ± s.e.m.; *n* = 4 experiments; two-tailed *t* test. Right: Representative images of terminal parasites +/−Shld1. Scale bar: 5 µm. **h** Top: Schizont and ring-stage parasites monitored by flow-cytometry following induction of *Pf*PP1-DD knockdown (+/−Shld1) late in the IDC. Bottom: DNA content in the parallel samples, with addition of E64 (50 µM). Mean of 2 technical replicates. Representative of 2 experiments. **i** Nuclear centers following *Pf*PP1-DD knockdown at 44 hpi, as in **g**. Mean ± s.e.m.; n = 4 experiments; two-tailed *t* test. Scale bar: 5 µm. **j** Egress and egress-to-invasion following induction of partial *Pf*PP1-DD knockdown at sublethal doses of Shld1. Mean ± s.e.m.; *n* = 4 experiments; two-tailed *t* test. Source data are provided as a Source Data file.

To investigate *Pf*PP1-mediated regulation of the IDC and parasite proliferation, we initiated a reverse genetic analysis. With a transgenic *P. falciparum* line expressing a triple-hemagglutinin (HA_3_) tag at the 3′-end of the endogenous *pfpp1* gene (Supplementary Fig. 1b, c), we found that *Pf*PP1 protein is expressed throughout development, with evidence for upregulation in the latter half of the 48-h IDC (Fig. 1a; Supplementary Fig. 1d). In agreement with cell biological analysis in the rodent malaria parasite *Plasmodium berghei*^19^ and subcellular fractionation in *P. falciparum*^21^, we observed by immunofluorescence analysis *Pf*PP1 near the nucleus and in the parasite cytoplasm throughout the IDC (Supplementary Fig. 1e). Cell cycle stage-dependent compartmentalization of PP1 has been reported in lower eukaryotes, consistent with distinct functions for the enzyme^22^. To assess the function of *Pf*PP1 in blood-stage proliferation, we generated a transgenic line of *P. falciparum* for inducible knockout of the *pfpp1* gene (*pfpp1-iKO*), based on the dimerizable Cre recombinase (DiCre) system (Supplementary Fig. 1b, f–h)^23,24^. Induction of *pfpp1-*knockout by rapamycin treatment early in the IDC, ~3–5 h post-invasion (hpi), results in strong reduction of *Pf*PP1 protein levels by ~30 hpi (Fig. 1b; Supplementary Fig. 1b, h). By following DNA replication through the IDC, we found that early-iKO of *pfpp1* delays parasite development before resulting in the accumulation of multinucleate schizont forms, blocked prior to egress (Fig. 1c, Supplementary Figs. 1i and 2a). Reverse genetic analysis by inducible knockout thus establishes the essentiality of *Pf*PP1 for asexual proliferation.

To investigate P*f*PP1 function at specific times through the IDC, we used a transgenic *P. falciparum* line for conditional knockdown (Supplementary Fig. 3a–c). Knockdown of *Pf*PP1 fused to a Destabilization Domain (DD)-tag, induced through depletion in culture of the DD-stabilizing small molecule Shield-1 (Shld1)^25–27^, confirms essentiality of the enzyme to blood-stage parasites (Fig. 1d, e; Supplementary Figs. 2b and 3d, e). In this transgenic line, we also observed *Pf*PP1 protein throughout the IDC, with expression more pronounced at later stages (Supplementary Fig. 3f). To map the time of function of *Pf*PP1, we induced knockdown at different stages of the IDC and measured ensuing DNA replication. We measured phenotypes with knockdown induced at the immature ring stage preceding the growth phase (4 hpi), at trophozoites before the onset of DNA replication (22 hpi), and early in schizogony (33 hpi) (Fig. 1f). With destabilization of *Pf*PP1-DD in rings, we observed substantial defects in DNA replication (Fig. 1f; Supplementary Figs. 2c and 3g). Knockdown induced in trophozoites permits DNA replication (Fig. 1f; Supplementary Figs. 2c and 3h) but results in parasites with reduced numbers of nuclear centers (Fig. 1g; Supplementary Fig. 3i), suggesting defects in nuclear division. Electron microscopy of *Pf*PP1-DD knockdown parasites indicates failure to complete the terminal mitosis and cytokinesis step of the IDC (Supplementary Fig. 3j), consistent with cell cycle-regulatory functions for *Pf*PP1 conserved with non-parasitic eukaryotes^10,11,28^. Defects upstream of cytokinesis with *Pf*PP1-DD knockdown are supported by immunofluorescence analysis: we observe that the inner membrane complex (IMC, antigen *Pf*GAP45) that separates replicated, intracellular parasites fails to form (Supplementary Fig. 3k).

To map functions for *Pf*PP1 late in the IDC, we induced knockdown in schizonts (~44 hpi), revealing an acute requirement for the phosphatase for egress after complete DNA replication and nuclear segregation (Fig. 1h, i; Supplementary Fig. 2c). Knockdown elicits a complete block in host cell egress and erythrocyte re-invasion (Fig. 1h; Supplementary Figs. 2d and 3l, m). Partial knockdown late in the IDC elicits sublethal defects in egress without additional defects observed in the further transition to invaded erythrocytes (egress-to-invasion) (Fig. 1j; Supplementary Fig. 2d), suggesting a specific function in egress.

### Requirement of P*f*PP1 at an early step of egress

**Fig. 2:**
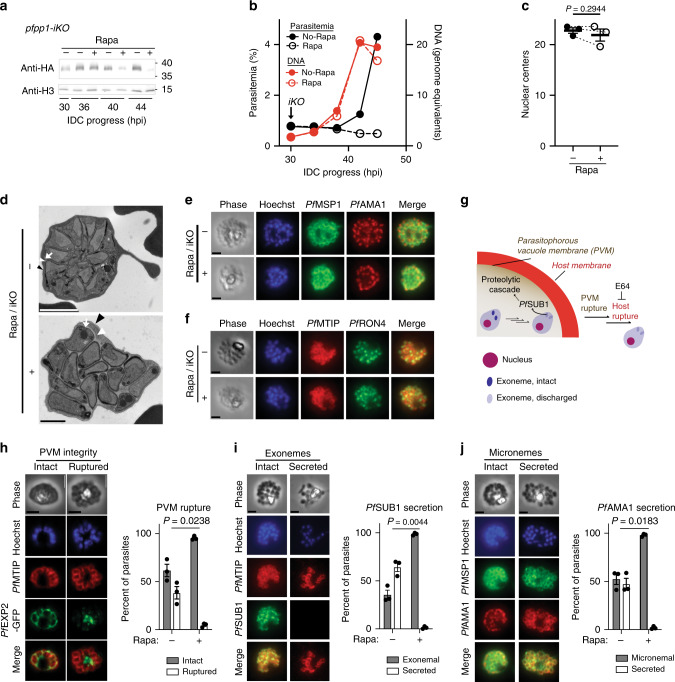
P*f*PP1 function at an early step of parasite egress from erythrocytes. **a** *Pf*PP1-HA_3_ expression +/− Rapa-mediated iKO of *pfpp1* at 30 hpi, assessed by immunoblot. Representative of 2 experiments. Molecular mass in kDa. **b** Parasitemia and DNA synthesis following iKO of *pfpp1* at 30 hpi, as in Fig. 1c. Mean of 3 technical replicates. Representative of 4 experiments. **c** Nuclear centers in terminally developed parasites following Rapa-mediated iKO of *pfpp1* at 30 hpi, as in Fig. 1. Mean ± s.e.m.; *n* = 3 experiments; two-tailed *t* test. **d** Electron microscopy of terminally developed *pfpp1*-*iKO* parasites treated +/−Rapa at 30-hpi. In both images, the different membranes are indicated as follows: erythrocyte (black arrowhead), PV (white arrowhead), and parasite (white arrow). Representative of 2 experiments. Scale bars: 2 µm (top), 1 µm (bottom). **e**, **f** Immunofluorescence analysis of the microneme antigen *Pf*AMA1 **e** or the rhoptry-neck antigen *Pf*RON4 **f** in terminally developed parasites +/−iKO of *pfpp1* at 30 hpi. The images also show the parasite plasma membrane marker *Pf*MSP1 **e** and the inner membrane complex marker *Pf*MTIP **f**. Scale bar: 2 µm. For both panels, representative of 2 experiments. **g** In a mature parasite, regulated secretion of *Pf*SUB1 from exonemes stimulates a proteolytic cascade leading to sequential rupture of the PVM and the erythrocyte host membranes. **h** PVM rupture at 45 hpi in *pfpp1-iKO* / *Pf*EXP2-GFP parasites treated +/− Rapa at 30 hpi. Left: immunofluorescence images of parasites with intact or ruptured PVMs. Right: Proportion of infected cells exhibiting PVM rupture. For **h**–**j**, mean ± s.e.m.; *n* = 3 experiments; two-tailed *t* test. In **h**–**j**, parasites were treated with E64 (50 µM) at 41 hpi; the completion of cytokinesis was assessed with the inner membrane complex marker *Pf*MTIP or the parasite plasma membrane marker *Pf*MSP1. Scale bars (**h**–**j**): 2 µm. **i** With +/− Rapa-treatment at 30 hpi in *pfpp1-iKO* parasites, quantification of *Pf*SUB1 secretion from exonemal compartments (loss of punctate fluorescence in images at left), as in **h**. **j** Assessment of *Pf*AMA1 secretion from micronemes, as in **h** and **i**. Source data are provided as a Source Data file.

The effects of late *Pf*PP1-DD knockdown are recapitulated in the *pfpp1-iKO* line with induction of Rapa-mediated iKO later in the IDC (30 hpi, Supplementary Fig. 4a), resulting in depletion of *Pf*PP1 protein at the late schizont stage (Fig. 2a). Late iKO blocks passage to new erythrocytes without defects in DNA replication or nuclear segregation (Fig. 2b, c; Supplementary Figs. 2a and 4b). Electron microscopy shows that parasites having undergone late *pfpp1-iKO* display gross morphology typical of maturation, including intact erythrocyte membranes, parasitophorous vacuoles that house parasites, and individual parasite cells physically distinguished by plasma membranes indicating the completion of cytokinesis (Fig. 2d). Immunofluorescence microscopy to image markers for the parasite plasma membrane (antigen *Pf*MSP1) and the underlying IMC (antigen *Pf*MTIP1) confirms that iKO of *pfpp1* does not perturb cytokinesis and segregation of these structures into replicated parasites (Fig. 2e, f; Supplementary Fig. 4c, d). Immunofluorescence shows also that secretory organelles utilized for invasion, micronemes (antigen *Pf*AMA1), and rhoptry necks (antigen *Pf*RON4), form normally with iKO of *pfpp1* (Fig. 2e, f). In the *Pf*PP1-DD line, immunofluorescence and electron microscopy show that parasites induced for knockdown late in the IDC undergo cytokinesis (IMC antigen *Pf*GAP45) and form normal rhoptries (antigen *Pf*RhopH3) (Supplementary Fig. 4e–g).

To initiate egress from erythrocytes at the end of the IDC, parasites secrete the protease *Pf*SUB1 in a regulated fashion from exoneme organelles into the lumen of the parasitophorous vacuole and activate a proteolytic cascade for sequential rupture of the vacuolar (PVM) and host cell membranes^29–31^ (Fig. 2g). To permit assessment of PVM rupture, we tagged the endogenously expressed PVM protein *Pf*EXP2 at the C-terminus with GFP^32^ in the *pfpp1-iKO* background (Supplementary Fig. 4h–j). Late in the IDC, *Pf*EXP2-GFP in intact PVMs presents intraerythrocytically as a circular label around the parasites or between replicated parasites, while rupture of the PVM can be observed by the appearance of fluorescent membrane fragments in parasites treated with E64 to prevent host cell rupture (Fig. 2g, h)^32^. We used labeling by *Pf*EXP2-GFP to test the requirement for *Pf*PP1 in PVM rupture, finding that late induction of iKO blocks the process in virtually all parasites examined (Fig. 2h). We further used immunofluorescence analysis to assess *Pf*SUB1 release required to initiate PVM rupture^31^, finding that rapamycin-treated *pfpp1-iKO* parasites fail to secrete the protease from exonemes (Fig. 2i). We observed that discharge of micronemes is also blocked by *pfpp1-iKO* (Fig. 2j). Our findings indicate an essential function for *Pf*PP1 at an early step of egress, following merozoite development but upstream of discharge of the specialized parasite organelles carrying proteases and other factors utilized for rupture of surrounding membranes.

### Identification of P*f*PP1 substrates by phosphoproteomics

**Fig. 3:**
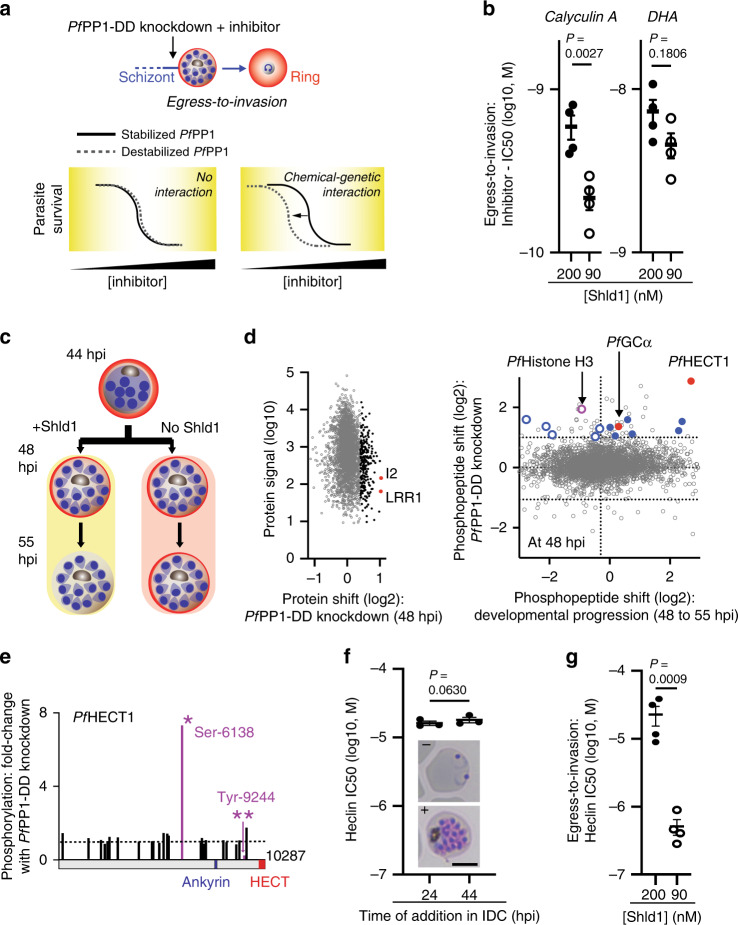
P*f*PP1 regulation of a HECT E3 protein-ubiquitin ligase for egress. **a** Chemical-genetics of *Pf*PP1-DD. We assessed functional interactions between inhibitor-sensitive processes and *Pf*PP1 from shifts in chemical sensitivity induced by knockdown of the phosphatase. **b** The sensitivities (IC50s) of *Pf*PP1-mediated egress-to-invasion to calyculin A and dihydroartemisinin (DHA) at 200 or 90 nM Shld1. Mean ± s.e.m.; *n* = 4 experiments; two-tailed *t t*est. **c** Scheme for phosphoproteomic analysis of *Pf*PP1-DD knockdown late in the IDC, with samples obtained at 48- and 55-hpi. **d** Left: At 48 hpi, signal intensities of individual proteins and shifts with *Pf*PP1-knockdown. *Pf*I2 and *Pf*LRR1 are indicated in red, with the top 5% of upregulated proteins indicated in black. Right: For all phosphopeptides detected in late-stage parasites, a plot of changes in levels with *Pf*PP1-DD knockdown at 48-hpi (*y-*axis) versus changes with development from 48 to 55-hpi in parasites on-Shld1 (x-axis). Thresholds for twofold increased and decreased phosphorylation (log_2_ = 1, *y*-axis) with knockdown are indicated. Upregulated phosphopeptides from gene products increased in transcription at the schizont-stage^16^ are colored; phosphopeptides in the upper-right quadrant (developmental progression threshold: median value, *x-*axis) least likely to be affected by secondary, developmental-progression defects (Supplementary Note 1) are indicated with filled circles. The phosphorylation site from *Pf*Histone H3 is purple; the sites from *Pf*HECT1 and *Pf*GCα are in red. Representative of 1 experiment. **e** Schematic of the predicted domains of *Pf*HECT1 protein. We show all phosphosites detected in our study with magnitude of change with *Pf*PP1-DD knockdown at 48 hpi as in **d**. The most increased (Ser-6138) and decreased phosphorylation sites (Tyr-9244) are indicated with symbols [*] and [**], respectively. **f** Sensitivity of *Pf*PP1-DD parasites on-Shld1 (0.5 µM) to heclin administered at the midpoint (24 hpi) or late in the IDC (44 hpi), determined from erythrocyte re-invasion. Mean ± s.e.m.; *n* = 3 experiments; two-tailed *t* test. Representative images of parasites at 55-hpi +/− heclin administration (100 µM) at 44 hpi, are shown. Scale bar: 5 µm. **g** The sensitivity of *Pf*PP1-mediated egress-to-invasion to heclin, as in **b**. Mean ± s.e.m.; *n* = 4 experiments; two-tailed *t* test. Source data are provided as a Source Data file.

Our reverse genetic analysis establishing the function of *Pf*PP1 at egress maps a factor typically associated with the conventional cell cycle of eukaryotes to the post-replicative stage of the IDC. To test if dephosphorylation of protein substrates accounts for the requirement for *Pf*PP1 in parasites late in the IDC, we implemented a chemical-genetic approach (Fig. 3a)^33^ to measure functional interaction between *Pf*PP1 and calyculin A, an active-site inhibitor of eukaryotic PP1^34^. We found that knockdown significantly increases the sensitivity of egress-to-invasion of parasites to calyculin A but not to the control antimalarial drug dihydroartemisinin (DHA) (Fig. 3b; Supplementary Fig. 2d), supporting a role for *Pf*PP1 phosphatase activity at this step. We found that *Pf*PP1-mediated intraerythrocytic development is also sensitive to calyculin A (Supplementary Figs. 2c and 5a), suggesting a requirement for protein dephosphorylation during this phase of host cell infection.

To identify potential substrates of *Pf*PP1 and probe regulation of parasites in the late IDC, we carried out a global phosphoproteomic analysis of *Pf*PP1-DD function spanning a period in the IDC from post-replication through egress. Following a restricted period (1.5 h) of parasite re-invasion from schizont-stage *Pf*PP1-DD parasites into new erythrocytes to generate synchronized cultures, we induced knockdown at 44 hpi and collected samples 4 and 11 h thereafter (Fig. 3c). We identified a total of 4720 phosphorylation sites from 1170 phosphoproteins, indicating phosphorylation of ~1/3 of all *P. falciparum* proteins we detected in late-stage parasites (Fig. 3d; Supplementary Data 1–3). We detected a comparable or greater number of phosphorylation sites than reported in other phosphoproteomic studies of late-stage *P. falciparum*^8,35–38^. Our dataset thus provides a comprehensive view of phosphorylation events with tight time-resolution through the course of egress. At the earliest timepoint following induction of *Pf*PP1-DD knockdown, there are minimal changes in either the global proteome or phosphoproteome between Shld1-supplemented and knockdown samples, indicating the absence of widespread global changes that may complicate assessment of specific phosphatase functions (Fig. 3d; Supplementary Note 1; Supplementary Fig. 5b–e).

*P. falciparum* homologs of established PP1 regulators for cell cycle progression^9,10,39–41^, the nuclear protein sds22 (in parasites, termed LRR1 for leucine-rich repeat protein 1^21^) and inhibitor-2 (I2)^20^, are among the two most strongly increased factors in protein expression upon *Pf*PP1-knockdown (Fig. 3d). Perturbed expression of these regulators may indicate a conserved function mediated by *Pf*PP1, while an increase in factors for glycolysis and the pentose phosphate pathway identified by gene ontology analysis suggest association with a parasite proliferative state (Supplementary Table 1)^42^. At 48 hpi, we observe by both phosphoproteomics and separately by immunoblot analysis increased phosphorylation, by up to ~5-fold, of Ser-29 of *Pf*Histone H3 (Fig. 3d; Supplementary Fig. 5f), homologous to Ser-28 in the human ortholog studied as a phosphorylation site targeted by PP1^43^. We thus observe among both putative regulators and substrates evidence for conserved PP1 activity in *P. falciparum*.

### P*f*PP1 regulation of a HECT E3 ligase for egress

The 50 proteins indicated by the 60 phosphopeptides increased >2-fold in phosphorylation upon *Pf*PP1-DD knockdown (Fig. 3d, Supplementary Data 3) include chromatin factors (histone H3 variant, histone deacetylase 1, chromodomain-binding protein), transcription factors from the AP2 family, and vacuolar-protein-sorting family members (VPS11 and VPS18). To focus on potential substrates for essential *Pf*PP1 function at egress, we identified gene products that specifically increase in transcriptional expression late in the IDC (Fig. 3d). The top hit from our phosphoproteomic screen, based on magnitude of increase in phosphorylation with *Pf*PP1-knockdown, is a previously uncharacterized, late IDC-stage protein carrying a ~300-amino acid HECT E3 protein-ubiquitin ligase domain at the C-terminus (PF3D7_0628100) (Fig. 3d, e; Supplementary Fig. 5g). In addition to the highly upregulated phosphorylation site (Ser-6138), knockdown of *Pf*PP1-DD also reveals a strongly downregulated site (>4-fold, Tyr-9244); the protein also contains a predicted site for interaction with *Pf*PP1^18^ near the HECT domain (Fig. 3e; Supplementary Fig. 5g). At >10,000 amino acids, the protein is the largest in the *P. falciparum* proteome.

The protein, which we name here *Pf*HECT1, is the single HECT domain-containing protein among four in *P. falciparum* to become increased in expression late in the IDC (Supplementary Fig. 5g). To test for specific HECT activity in parasites late in the IDC, we probed for susceptibility to the small molecule heclin (Fig. 3f). Heclin was identified as a broad-spectrum inhibitor of mammalian HECT enzymes, with biophysical studies suggesting that direct binding by the compound interferes with transfer of ubiquitin from the E2 adaptor protein to an active-site cysteine in the E3 ligase^44^. In *Pf*PP1-DD parasites stabilized with Shld1, we established the antimalarial activity of heclin toward parasites, with short (~4 h) and longer periods of exposure (~24 h) preceding egress exhibiting similar potency of inhibition toward establishment of ring-stage parasites (IC50 ~20 µM), suggesting major activity in late schizonts (Fig. 3f; Supplementary Fig. 2d). While *Pf*PP1-DD destabilization does not augment DNA replication defects induced by heclin (Supplementary Figs. 2c and 5h), knockdown in parasites late in the IDC strongly increases susceptibility of egress-to-invasion to the inhibitor, reducing the IC50 by >100-fold to submicromolar levels (Fig. 3g; Supplementary Figs. 2d and 5i). An assessment of schizont rupture shows that inhibition of egress by heclin is also strengthened by *Pf*PP1-DD destabilization (Supplementary Figs. 2d and 5j). Phosphoproteomic analysis (Fig. 3d) with chemical-genetic analysis (Fig. 3g) indicates a critical function for *Pf*PP1 in activation of *Pf*HECT1-mediated E3 protein-ubiquitin ligase activity for egress.

### P*f*PP1 regulation of cGMP synthesis

**Fig. 4:**
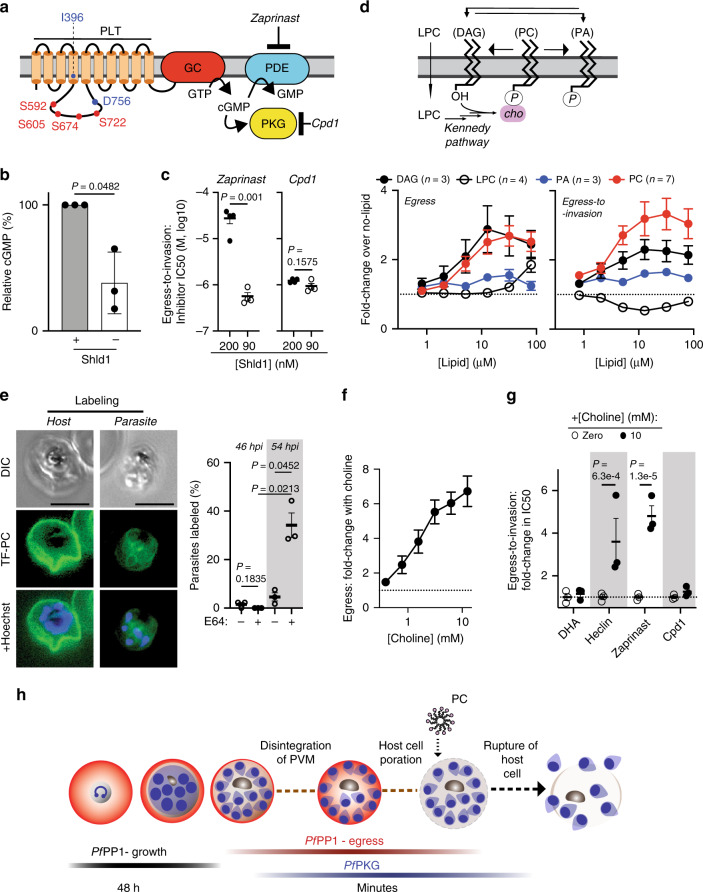
P*f*PP1 regulation of cGMP and signaling by extrinsic phosphatidylcholine. **a** *Pf*PP1-regulated phosphorylation and cGMP-based signal transduction initiated by *Pf*GCα. We indicate *Pf*PP1-regulated sites (red) and signature sequences for phospholipid transporter (PLT) activity (purple), Ile-396 and Asp-756 (Supplementary Fig. 6a). cGMP-phosphodiesterase (PDE) and *Pf*PKG, as well as inhibitors of both targets, are depicted. **b** Relative cGMP at 47.5 hpi in *Pf*PP1-DD-intact (0.5 µM Shld1) or knockdown parasites. Mean ± s.e.m.; *n* = 3 experiments; two-tailed *t* test. **c** The sensitivity of *Pf*PP1-mediated egress-to-invasion to zaprinast (left) or Cpd1 (right). Mean ± s.e.m.; *n* = 4 experiments; two-tailed *t* test. **d** Top: Phospholipids at the parasite plasma membrane with potential routes of interconversion. Extracellular LPC crosses the plasma membrane, providing substrate for endogenous biosynthesis of PC via the Kennedy Pathway^59, 60^. Bottom: Stimulation of egress (left) or egress-to-invasion (right) by supplemented lipids in late IDC *Pf*PP1-DD parasites with partial destabilization (100 nM Shld1), expressed in terms of fold-change relative to no-lipid conditions (mean ± s.e.m., number of experiments indicated in plot). **e** Labeling of the parasites by PC. Left: Representative images of late-stage *Pf*PP1-DD-infected erythrocytes (0.5 µM Shld1 and 50 µM E64, 54 hpi) labeled by TF-PC at the erythrocyte membrane or at the plasma membranes of internal parasites. Nuclei are indicated by Hoechst. Scale bar: 5 µm. Right: The proportion of infected erythrocytes with labeled parasites at the indicated timepoints, ±E64. Mean ± s.e.m.; *n* = 3 experiments; two-tailed t test. **f** Stimulation of *Pf*PP1-mediated egress with supplementation of choline, as in **d**. Mean ± s.e.m.; *n* = 4 experiments. **g** Modulation of *Pf*PP1-regulated cellular processes by supplemented choline. The fold-change in IC50 with additional choline in *Pf*PP1-DD parasites in 150 nM Shld1 is indicated. Mean ± s.e.m.; *n* = 3 experiments; multiple two-tailed t tests (false discovery rate, 1%). **h** A model for the function of *Pf*PP1. Following regulation of growth during development, *Pf*PP1 is essential for the egress program upstream of cGMP-activated *Pf*PKG and disintegration of the PVM. PC enters host cells following natural permeabilization of the erythrocyte membrane, acting on exposed parasites to stimulate egress. Source data are provided as a Source Data file.

Among other late IDC-stage *P. falciparum* genes showing increased phosphorylation with *Pf*PP1-knockdown is a guanylyl cyclase (GC) domain-containing protein encoded by the gene PF3D7_0381400 (Fig. 3d; Supplementary Fig. 6a), required to synthesize cyclic guanosine monophosphate (cGMP) to stimulate the downstream effector *Pf*Protein Kinase G (*Pf*PKG) and egress from infected erythrocytes (Fig. 4a)^31,45,46^. Termed GCα, the protein is present across apicomplexan parasites, and in *Toxoplasma gondii* is also essential for egress^47–50^. Given the function we identified for *Pf*PP1 in egress and phosphoregulation of *Pf*GCα, we measured the effect of *Pf*PP1-DD knockdown on cellular cGMP levels, finding that destabilization of the phosphatase enzyme reduces the second messenger in schizont-stage parasites by ~2-fold (Fig. 4b). To understand the functional significance of *Pf*PP1 for cGMP-stimulated egress, we implemented chemical-genetics to test for functional interaction with components of signal transduction related to the second messenger. To induce cGMP in parasites late in the IDC, we used zaprinast, a phosphodiesterase (PDE) inhibitor^31,51^ (Fig. 4a). The susceptibility of egress-to-invasion of *Pf*PP1-DD parasites to zaprinast becomes sharply increased with knockdown: IC50 values drop by as much as ~400-fold to submicromolar levels (Fig. 4c; Supplementary Figs. 2d and 6b). We found that zaprinast inhibition is mitigated by the *Pf*PKG inhibitor Compound-1 (Cpd1)^45^ (Supplementary Figs. 2d and 6c), consistent with the interpretation that toxicity of the PDE inhibitor requires activation of the well-established downstream effector of cGMP signaling in *Plasmodium* parasites. In contrast to zaprinast, functional interaction of *Pf*PP1-DD with Cpd1 is weak, with measurements of egress-to-invasion showing no chemical-genetic interaction (Fig. 4c). Parasite egress is more sensitive to Cpd1 with *Pf*PP1-DD knockdown, though much less than the shift in sensitivity observed with zaprinast (Supplementary Figs. 2d and 6d). Our analysis of parasites late in the IDC indicates regulation by *Pf*PP1 of guanylyl cyclase activity, upstream of activation of *Pf*PKG for numerous egress and invasion processes^37,52^. We note that in parental, genetically unmodified parasites, Shld1 does not affect sensitivity to calyculin A, heclin, or zaprinast (Supplementary Figs. 2d and 6e), indicating that the chemical-genetic interactions we have observed in this study with *Pf*PP1-DD knockdown result from impairment of phosphatase function.

### Phosphatidylcholine as an extrinsic signal for egress

*N*-terminal to the GC domain, GCα contains a putative P4-ATPase phospholipid transporter (PLT) domain predicted to translocate phospholipids from the exoplasmic to the cytoplasmic face of membranes. The fusion of PLT with the GC domain is a structure found only in alveolates^53^, and we observed that *Pf*PP1-responsive phosphorylation sites (1.4–2.6-fold upregulation with knockdown) cluster in a parasite-specific cytoplasmic loop of PLT containing a catalytic site predicted essential for P4-ATPase activity (Fig. 4a; Supplementary Fig. 6a; Supplementary Data 3). Mutational analysis in *T. gondii* GCα indicates the requirement for fusion of the PLT and GC domains as well as ATP-dependent catalysis by the PLT^47,48^, raising the possibility of a role for phospholipids in function of the protein, potentially for egress. To directly assess involvement of phospholipids in *Pf*PP1-regulated egress, we administered synthetic phospholipids to parasites late in the IDC. We tested phosphatidic acid (PA), known to stimulate host cell egress in *T. gondii* when added extracellularly^47^ and also through endogenously synthesized forms resulting from intracellular phosphorylation of the neutral lipid diacylglycerol (DAG)^54,55^. We also tested phosphatidylcholine (PC), the major species of phospholipid in host serum at concentrations of ~1–2 mM^56,57^. In parasites with intact *Pf*PP1-DD (300 nM Shld1), neither phospholipid influences egress (Supplementary Figs. 2d and 6f). In parasites with partially destabilized *Pf*PP1-DD, however, PA stimulates egress by up to ~1.5-fold and PC up to ~3-fold, with effects observed as low as 5 µM phospholipid (Fig. 4d; Supplementary Fig. 2d). An effect for PA might be explained by the proposal that the molecule provides one of multiple signals required for productive egress, acting on targets to activate microneme secretion and parasite motility, downstream of cGMP and phosphatidylinositol signaling^52,55,58^. We indeed observed that knockdown of *Pf*PP1-DD late in the IDC increases susceptibility to the DAG-kinase inhibitor R59022 that restricts endogenous PA synthesis (Supplementary Figs. 2d and 6g), consistent with a pro-egress function for the phospholipid in *Plasmodium* parasites^54^.

A role for PC in egress has not been described. We found that extracellular DAG stimulates egress to similar levels as PC with partial *Pf*PP1-DD destabilization, suggesting convergent targets or efficient conversion between the two lipids upon incorporation into the parasite from the extracellular medium (Fig. 4d; Supplementary Fig. 2d). Merozoites released by PC infect erythrocytes with efficiency comparable to the magnitude of stimulated egress (Fig. 4d; Supplementary Fig. 2d), supporting a physiological function for the phospholipid in invasive parasites near the natural timing for host cell rupture. Compared to PC, DAG yields merozoites with invasiveness reduced by ~30% (Fig. 4d; Supplementary Fig. 2d). Lysophosphatidylcholine (LPC), which drives parasite PC biosynthesis via the Kennedy Pathway^59,60^, stimulates egress weakly in comparison to direct administration of the phospholipid (Fig. 4d); and glucose supplementation to increase endogenous DAG^59,61^ does not stimulate egress (Supplementary Figs. 2d and 6h).

While erythrocyte membranes housing developing *P. falciparum* parasites block access to free PC^59^, host cells abruptly become permeable to extracellular solutes in the seconds to minutes preceding egress^30,32,62^, presenting a route for direct interaction between parasites and circulating phospholipids. We thus tested accessibility of parasites to PC during egress, using a fluorescent analog (labeled with TopFluor, TF). In most conditions we observed that TF-PC marks only the outer membranes of erythrocytes housing multinucleate schizonts (Fig. 4e). We observed clear labeling of parasites within infected erythrocytes, however, at a later timepoint when parasites have naturally entered the egress program and acquired host cell permeability (Fig. 4e). The intact schizont with permeable erythrocyte membrane is a transient state prior to egress, stabilized by E64-treatment^30,62,63^. We conclude that circulating PC accesses parasites when host cells become permeable to the extracellular environment, shortly before natural host cell rupture.

Endogenous PC synthesis is promoted with the addition of the precursor choline^59,60^. We found that choline stimulates egress in parasites with partially destabilized *Pf*PP1-DD; albeit in contrast to PC, only at non-physiological serum levels^64^ (Fig. 4f; Supplementary Fig. 2d). Identical, high concentrations of choline do not influence egress by parasites with intact *Pf*PP1-DD (300 nM Shld1) (Supplementary Figs. 2d and 6i). We found that high choline decreases susceptibility of parasites specifically to heclin and zaprinast (Fig. 4g; Supplementary Fig. 2d). Our analysis suggests that a PC signal for egress interacts with cell-intrinsic pathways regulated by *Pf*PP1.

## Discussion

We have functionally characterized *Pf*PP1 through the blood-stage IDC of malaria parasites. In addition to potential conserved roles in development, *Pf*PP1 is essential for egress. At the pre-erythrocytic liver-stage, analysis by other researchers shows the non-essentiality of *Plasmodium* PP1 for intrahepatocytic development, though a function for the protein phosphatase in egress into the bloodstream was not assessed^65^. Previous studies of *Plasmodium* regulation of egress in erythrocytic parasites have focused on the second messenger-responsive protein kinases *Pf*PKG and *Pf*Calcium Dependent Protein Kinase 5^26,31,37,45,66^, neither of which is implicated as a substrate of *Pf*PP1 in our study. Our functional analysis of *Pf*PP1 instead delineates a regulatory mechanism upstream of stimulation of second messengers. *Pf*PP1 targets a limited set of proteins previously noted as *Pf*PKG-regulated^37^ (Supplementary Data 3), including GCα, raising the possibility of negative feedback for regulation of cGMP synthesis. *Pf*Schizont Egress Antigen 1^67^ is among the proteins we found with *Pf*PP1-regulated sites (Supplementary Data 3), perhaps indicating phosphoregulation also of this factor for egress.

We found that *Pf*PP1 regulates *Pf*HECT1 (Supplementary Fig. 7a), demonstrating a role for ubiquitination in egress alongside well-established pathways for phosphorylation and proteolysis. For *Pf*HECT1, *Pf*PP1-mediated phosphorylation in the interior of the polypeptide may indicate a mechanism for regulation of the distal enzymatic domain, e.g. by autoinhibition^68^ or by trans-acting factors^69^ as has been described for other HECT-family E3 ligases.

At multiple stages of the *Plasmodium* lifecycle, signaling by cGMP is utilized for colonization of new host niches, and studies indicate that a specific timing of activation and level of the second messenger are critical for infectivity^31,70–72^. With the phosphoproteomic analysis, the requirement of *Pf*PP1 at the blood-stage for induction of cellular cGMP in schizont-stage parasites leads us to hypothesize that *Pf*PP1 stimulates synthesis of the cyclic nucleotide by *Pf*GCα (Supplementary Fig. 7a). Regulation of cGMP by *Pf*PP1 may arise from direct interaction of the phosphorylated PLT with the GC domain and/or result from phospholipid translocation regulated by phosphorylation of the PLT. Sensitivity of *Pf*PP1-mediated egress to zaprinast shows that hyperactivation of cGMP synthesis blocks egress, an outcome that we propose reflects a just-in-time regulatory structure controlled by *Pf*PP1. Specifically, we suggest that *Pf*PP1 regulates cGMP induction in coordination with additional processes for egress, with aberrant stimulation of *Pf*PKG preceding other functions resulting in unproductive activation of the egress machinery (Supplementary Fig. 7b).

We discovered PC as a parasite-extrinsic factor that stimulates egress from erythrocytes, distinct from the extracellular LPC signal in *P. falciparum* that suppresses differentiation to sexual-stage forms early in schizogony^59^. This finding supports a model wherein *Pf*PP1 dephosphorylates the phospholipid transporter domain of *Pf*GCα to promote translocation of PC across the parasite plasma membrane and stimulate egress. PC may act as a trigger for cGMP synthesis or as a concurrent signal for egress (Supplementary Fig. 7a). Our study also demonstrates the accessibility of exogenous PC to the host and parasite plasma membranes and ability to influence egress at a late step following host cell poration (Fig. 4h), consistent with function for cGMP-activated *Pf*PKG in free merozoites for erythrocyte invasion^37^. With *Pf*PP1 (Fig. 2), *Pf*PKG is essential also at an earlier step of egress for disruption of the PVM preceding deterioration of the host cell membrane^31,32,63^. In the absence of host cell permeability at an early stage of egress, intraerythrocytic sources may well provide PC for stimulation of cGMP. Indeed, poration of the PVM preceding *Pf*PKG activation has been reported^63^, indicating a potential source of PC accessible to intracellular merozoites.

Our functional analysis of *Pf*PP1 at egress reveals a regulatory nexus for host cell passage at which cell-intrinsic pathways are coordinated with environmental signals to ensure release of invasive parasites into circulation and infection of new host cells.

## Methods

### Reagents and antibodies

Rapamycin (LC laboratories), E64 (Sigma-Aldrich, Cat. No E3132), dihydroartemisinin (Sigma-Aldrich, Cat. No. D7439), calyculin A (Sigma-Aldrich, Cat. No. C5552), heclin (Sigma-Aldrich, Cat. No. SML1396), zaprinast (MP Biomedicals, Cat. No. ICN15693180), were each prepared in DMSO. Choline chloride (Sigma-Aldrich, Cat. No. 7527) was prepared in water. DAG and all phospholipids with mono-unsaturated diacylglycerol backbone (16:0, 18:1) from Avanti [PA, Cat. No. 840857; PC, Cat. No. 850457; TopFluor-PC, Cat. No. 810281; and DAG, Cat. No. 800815] were solubilized at 1 or 5 mM in 100% methanol, except PC (water: ethanol: methanol; 1:1:2). LPC (Cat. No. 855675) was solubilized at 200 mM in a 1:1 mixture of ethanol and water. Compound-1 (DMSO-based) was a gift from Dr. Jeffrey Dvorin (Boston Children’s Hospital). Dilutions and sources for antibodies for immunoblot or immunofluorescence analysis are as follows: rabbit anti-GAP45 (1:5000, gift from Dr. Julian Rayner, Wellcome Trust Sanger Institute, Hinxton, UK); mouse anti-RhopH3 (1:200, gift from Jean-Francois Dubremetz); rabbit α-MTIP (1:500, gift from Tony Holder, The Francis Crick Institute, UK), mouse α-MSP1.19 (1:1000, gift from M. Blackman, The Francis Crick Institute, UK), mouse α-RON4 [1:200, home-made^73^], mouse α-SUB1 (1:2, gift from M. Blackman), rabbit α-AMA1 (1:1000, gift from M. Blackman); rabbit anti-histone H3 (1:10,000, Abcam ab1791); rat anti-HA antibody 3F10 (1:1000, Roche Cat. No. 11867423001); anti-phospho S28 histone H3 antibody (1:1,000, Abcam Cat. No. ab5169); rabbit anti-*Pf*aldolase-HRP (Abcam, 1:2000); and mouse anti-GFP (Roche, 1:1000). The secondary antibodies used for IFA were Alexa-Fluor 488 and 594-conjugated antibodies against mouse, rat, or rabbit IgG diluted as recommended by the manufacturer (Invitrogen A21208, A11012, A11001, A11008, A21209, and A11005). Shield-1 (Shld1) was synthesized as described^25,74^ and dissolved to 1 mM stock concentration in absolute ethanol before use.

### Plasmids

Primers for PCR amplification and verification of transgenesis in *P. falciparum* are shown in Supplementary Table 2. The plasmid for generation of the transgenic parasites *pfpp1*-*ha*_*3*_ was obtained using the plasmid pLN-PP1-HA_3_-loxP. To generate the plasmid pLN-PP1-HA_3_-loxP, we first introduced between the BamHI and HpaI sites in pLN-ENR-GFP^75^ a synthetic fragment with sequence for a triple-hemagglutinin tag (HA_3_) followed by a stop codon and a loxP site (IDT DNA), and multiple cloning sites upstream of the tag. The resulting plasmid pLN-PP1-HA_3_-loxP was further modified to target endogenous *pfpp1* with HA_3_ and loxP by introduction of a 5′ homology region for the gene (HR1, 682 bp of genomic DNA sequence for exons 2 and 3) fused to a recodonized synthetic fragment (IDT DNA) for exons 4 and 5. The PCR-amplified elements were ligated in a single reaction step by In-Fusion cloning (Clontech) upstream of the HA_3_ tag in XmaI and AfeI sites of pLN-HA_3_-loxP. The *pfpp1* 3′ homology region (HR2) carrying 440 bp of the *pfpp1* 3′-UTR was PCR-amplified and inserted by In-Fusion reaction 3′ of the loxP site between PstI and HpaI. The guide RNA sequence for targeting *pfpp1* near exon 3 was cloned into the BbsI sites in pDC2-cam-co-Cas9-U62-hDHFR (gift from Dr. M. Lee, Wellcome Sanger Institute). For subsequent engineering of a *pfpp1* conditional knockout in parasites, the pLN-PP1-loxPint plasmid was modified with introduction of the following elements ligated in 5′ to 3′ order between the BamHI and ApaI sites of pLN-ENR-GFP: PCR-amplified fragment of *pfpp1* encompassing 5′UTR and part of exon 1 (382 bp), a synthetic fragment for a recodonized 3′ sequence of exon 1 followed by the artificial loxPint (IDT DNA), and a PCR-amplified fragment of the 5′ end of *pfpp1* exon 2 (601 bp). The plasmid for the guide RNA targeting *pfpp1* near exon 1 was constructed as above. To tag endogenous *Pf*EXP2 with GFP, we generated plasmid pLN-*Pf*EXP2-GFP as described^32^. Two homology regions for the gene *pfexp2* were cloned in pLN-ENR-GFP on both sides of the GFP coding sequence: 549 bp of *pfexp2* 3′ coding sequence without the stop codon in frame with GFP, and 453 bp of *pfexp2* 3′UTR downstream of GFP. The *pfexp2* guide RNA sequence^32^ was cloned into the BbsI sites of pDC2-cam-co-Cas9-U62-hDHFR. Plasmid pAK8 for 3′-single-crossover HA-DD-tagging at endogenous *pfpp1* was constructed in the pJDD41 background^26^ with the PCR-amplified targeting fragment, amplified from *P. falciparum* D10 genomic DNA, ligated between the NotI and XhoI restriction sites. All plasmid sequences were verified before downstream applications. Plasmid pARL2-GFP is previously reported^76^.

### Parasite culture

D10 or 3D7 (Walter and Eliza Hall Institute), or p230p-based parasites^23^ were cultured continuously in human erythrocytes^77^ obtained from a commercial source (Research Blood, Boston) or anonymous donors from the French Bloodbank (Etablissement Français du Sang, France) under the approval number 21PLER2016-0103. We complied with all ethical regulations in use of human blood. Continuous culture was typically carried at 2–5%-hematocrit in RPMI-1640 (Sigma Aldrich, Cat. No. R6504) supplemented with HEPES, 25 mM; Albumax II, 4.31 mg ml^−1^ (Thermo Fisher Scientific), or 10% human serum; sodium bicarbonate, 2.42 mM; and gentamycin (20–25 µg ml^−1^). Parasites were cultured at 37 °C in hypoxic conditions (1–5% O_2_, 5% CO_2_, with N_2_ as balance) in modular incubator chambers. Parasites were transfected by electroporation^78^, and treated with WR99210 (2.5–5 nM) or blasticidin (2.5 µg ml^−1^). *Pf*PP1-DD transfectants were further selected for single-crossover integrants by cycles of on-drug and off-drug treatment^79,80^. All transgenic lines were cloned by limiting dilution and genotyped by PCR. Unless otherwise noted, all experiments with *Pf*PP1-DD parasites indicate a line constructed in the D10 background.

We synchronized parasites with heparin (100 units ml^−1^) to define restricted periods of invasion^81^. Alternatively, we isolated schizonts by magnetic affinity purification (MACs LS column, Miltenyi, fitted with a 23-gauge needle) or 70% Percoll cushion, and allowed invasion into uninfected erythrocytes for a defined period. Following invasion, we either added heparin to block further invasion, selectively lysed remaining schizonts by sorbitol treatment (5% w/v in double-distilled water), or isolated recently invaded rings from unruptured schizonts by magnetic affinity purification (MACs LS with 27-gauge needle).

### Statistical significance testing

We carried out all tests for statistical significance in Prism software (GraphPad). Unless otherwise stated, *P* values indicate the results of two-tailed, paired *t* tests. For multiple *t* tests, false discovery rate (FDR) calculation was implemented using the default two-stage step-up method of Benjamini, Krieger, and Yekutieli.

### Induction of *Pf*PP1-phenotypes through conditional expression

For assays, we induced parasites for either knockout of *pfpp1* [mock- (DMSO) versus Rapa, 10 nM] or knockdown of *Pf*PP1-DD [Shld1 (0.2–0.5 µM) versus ethanol vehicle]. In knockout parasites, we washed away Rapa 4 h following addition.

### Immunoblot analysis

For immunoblot analysis, we released parasites from erythrocytes with cold PBS containing 0.1–0.2% saponin, and boiled in SDS-PAGE sample buffer^80^. Following electrophoretic separation, proteins were transferred to a nitrocellulose membrane, and immunoblot analysis was carried out using the LI-COR system (Lincoln, USA), or the Chemidoc system (Bio-Rad).

### Microscopy

For quantitative assessment of nuclear centers in terminally developed *Pf*PP1-DD parasites [~54–60 hpi; +E64 (50 µM) since ~45 hpi], we collected ~500,000 infected cells onto glass slides by cytospin centrifugation, followed by fixation in methanol and staining with May-Grünwald-Giemsa. All infected cells encountered in visual fields by conventional light microscopy (>54 per sample) were counted. In the *pfpp1-iKO* line, we used immunofluorescence microscopy (see below) to identify segmented parasites that stained positive for the antigen *Pf*MTIP before counting nuclei stained with DAPI. For immunofluorescence^66^, thin smears were fixed in 4%-paraformaldehyde in PBS for 10 min at room temperature or overnight at 4 °C in a humidified chamber followed by extensive washing in buffer; permeabilized with 0.1%-Triton-X-100/PBS for 10 minutes at room temperature before further washing; blocked with 3% BSA/PBS for >1 h at room temperature or overnight at 4 °C; treated with primary antibody overnight at 4 °C; washed extensively before treatment with the appropriate Alexa-Fluor 488 or 594-conjugated secondary antibody for 1 h at room temperature; washed and prepared in DAPI-containing mounting solution for imaging. Images were taken with a Zeiss Observer Z1, Zeiss Axioimager Z2, or confocal Zeiss LSM880 equipped with an Airyscan module, and processed with Zen 2 blue edition software (Zeiss) or Fiji^82^. For immunofluorescence assays for PVM rupture or secretion of exoneme or microneme antigens, parasites were treated at 41 hpi with 50 µM E64 and smeared 4–5 h later for analysis.

For assessment of TF-PC labeling of *P. falciparum*, we treated synchronous (±1 h) *Pf*PP1-DD parasites (+0.5 µM Shld1) with or without E64 (50 µM) for ~7 h before image acquisition at the indicated timepoints. After evaporation of TF-PC on the surface of multiplate wells, we added parasites in standard media (2%-hematocrit) for a final concentration of fluorescent label of 100 µM. After ~30 min at 37 °C in standard culture conditions, cells were collected and stored at 4 °C until imaging carried out over the course of the next ~2 h. Just before imaging, parasites were spotted and mixed with Hoechst dye on coverslips (No. 1.5) pre-treated with Concanavalin-A (Sigma-Aldrich, Cat. No. C5275, 0.5 mg ml^−1^ in PBS; spread and dried at 37 °C for ~30 min) immediately before sealing by surface tension and dispersion of cell suspension with a glass slide. Images were acquired with a 63×-objective on the Zeiss Observer Z1, in the DIC, DAPI, and GFP channels. We scored 40–66 multinucleate, infected cells per condition to estimate internal labeling of parasites.

For transmission electron microscopy (TEM) of *pfpp1-iKO* cells, we directly added 25% glutaraldehyde (EM grade) to the culture medium to obtain a final concentration of 2.5%. After 10 min incubation at room temperature, we centrifuged the cells and resuspended the pellet in 20 volumes of cacodylate buffer (0.1 M) containing 2.5% glutaraldehyde and 5 mM CaCl_2_.The suspension was left 2 hours at RT before long-term storage at 4 °C in fixative until further processing. All the following incubation steps were performed in suspension, followed by centrifugation using a benchtop microcentrifuge. Cells were washed with cacodylate buffer and post-fixed with 1% O_s_O_4_ and 1.5% potassium ferrocyanide in cacodylate buffer for 1 h. After washing with distilled water, samples were incubated overnight in 2% uranyl acetate in water and dehydrated in graded series of acetonitrile. Impregnation in Epon 812 was performed in suspension on a rotary shaker for 1 h in Epon: acetonitrile (1:1) and 2 × 1 h in 100% Epon. After the last step, cells were pelleted in fresh epon and polymerized for 48 h at 60 °C. 70 nm sections were made with an ultramicrotome Leica UC7, contrasted with uranyl acetate and lead citrate and imaged by TEM on a JEOL 1200 EX. All chemicals were purchased from Electron Microscopy Sciences (USA).

TEM analysis of *Pf*PP1-DD parasites was carried out similarly, with some modifications. For fixation, 1 volume of suspended culture (>~5 µl packed cell volume) was supplemented with 1 volume of a 2x fixative solution (5% glutaraldehyde, 2.5% paraformaldehyde, 0.06% picric acid in 0.2 M cacodylate buffer, pH 7.4), spun briefly at 500 g, and stored at 4 °C before further processing. Following fixation, cells were washed in water, then maleate buffer before incubation in 2% uranyl acetate (1 h). Following washes in water, dehydration was done in grades of alcohol (10 min each at 50%, 70%, 90%, and 2 × 100%). The samples were then put in propyleneoxide for 1 h and infiltrated overnight in a 1:1 mixture of propyleneoxide and TAAB Epon (Marivac Canada Inc. St. Laurent, Canada). The following day, the samples were embedded in TAAB Epon and polymerized at 60 °C for 48 h. Ultrathin sections (about 60 nm) were cut on a Reichert Ultracut-S microtome, picked up on to copper grids stained with lead citrate and examined in a JEOL 1200EX or a TecnaiG² Spirit BioTWIN. Images were recorded with an AMT 2k CCD camera.

### Flow-cytometry and analysis

All measurements of parasitemia by flow-cytometry were carried out with staining of fixed cells with SYBR-Green I (Invitrogen) to distinguish DNA-containing parasitized erythrocytes from uninfected, enucleate erythrocytes^80,83–85^. Fixation of *Pf*PP1-DD cell suspension was carried out by addition of >3 volumes of PBS supplemented with paraformaldehyde (4% final concentration) and glutaraldehyde (0.0075-0.015% final concentration), followed by storage at 4 °C for >12 h before further washes in buffer. For quantitative measurements of cellular DNA, fixed cells were permeabilized with Triton-X-100 (0.1%) and RNase-treated (~0.3 mg ml^−1^) before staining^85^. For *pfpp1-iKO* parasites, cells were fixed by addition of an equal volume of PBS-paraformaldehyde (8%); fluorescence was measured with a BD FACS Canto I cytometer and 100,000 events were recorded per sample. For *Pf*PP1-DD parasites, flow-cytometry was carried out with a MacsQUANT 10 instrument (Miltenyi) on the FITC channel. We measured at least 20,000 cells per sample. All flow-cytometry data were analyzed with FlowJo software.

To calculate cellular DNA content, we used fluorescence measurements from uninfected erythrocytes (zero genomes), singly-infected rings (1 genome), and doubly-infected rings (2 genomes), to generate standard curves for translation of total fluorescence of an infected erythrocyte population into genome equivalents (Supplementary Fig. 2). In some measurements, we applied a background correction across all samples to subtract the contribution of parasite cells that did not advance into DNA replication by 48-hpi (i.e. in +Shld1-conditions). To calculate egress from parasites late in the IDC in an experiment, we used the remaining schizont populations measured at the end of an assay. We treated schizont levels in No-Shld1 conditions (or 50 nM Shld1, Supplementary Fig. 6f, i) as a measure of no-egress (zero), and levels with high-Shld1 as full egress (100%). We similarly calculated egress-to-invasion from parasites late in the IDC using ring-stage parasite levels. Fold-change in egress, as reported in Fig. 4, is the quotient of schizont levels at the end of the assay in the absence of chemical (numerator) to schizonts levels left in the presence of chemical (denominator).

### Measurement of cellular cGMP

*Pf*PP1-DD parasites (0.5 µM Shld1) were synchronized by magnetic affinity purification of schizonts followed by heparin treatment to define an invasion window of 4 h (see above). At 44 hpi (±2 h), we repeated magnetic affinity purification in the absence of Shld1, using a 27 G insulin needle for maximum recovery of schizonts. Following four washes in excess volume of RPMI to remove residual Shld1, schizonts were resuspended in media (~3.5−6.5 × 10^6^ cells ml^−1^), split with either 0.5 µM Shld1 or ethanol vehicle, and replaced in culture at 2 technical replicates per experimental condition. After 3.5 h, cells were centrifuged twice (930 × *g*, 2 min, room temperature) to extensively remove culture supernatant before snap-freezing in liquid nitrogen and long-term storage at −80 °C.

We used a competitive ELISA assay kit (Cayman Chemical Cat. No. 581021) to measure cGMP extracted from schizonts. We followed the manufacturer’s acetylation protocol following direct lysis of cells in 0.1 M HCl and appropriate dilutions into the commercial ELISA buffer. Following 14-18 h of incubation of plated samples with cGMP antiserum and tracer, and several washes, we carried out development with the colorometric Ellmans Reagent for ~1 h before absorbance measurements (λ, 405). Reported data are from measurements of samples corresponding to approximately 0.7–5 × 10^6^ schizonts (average of two technical replicates per experiment). Sample cGMP levels are estimated from logit transformed linear fits of measurements of provided cGMP standards.

### Proteomic and phosphoproteomic profiling

*Pf*PP1-DD parasites were synchronized as described above with MACs purification of schizonts and treatment with sorbitol (5% w/v in water) after 1.5 hours of invasion into uninfected erythrocytes to eliminate remaining schizonts and isolate freshly re-invaded rings. Approximately 10 × 10^10^ synchronous ring-stage *Pf*PP1-HA-DD parasites in Shld1 (0.3 µM) were cultured to 44 hpi with a preceding change of warm media at ~24 hpi, washed extensively and replated in warm (37 °C) complete-RPMI at ~5 × 10^6^ parasitized erythrocytes per ml of culture ±0.3 µM Shld1. At 48 hpi, for each Shld1 condition, 2 technical replicates each with or without Shld1 (~1 × 10^10^ parasitized erythrocytes per replicate) were centrifuged at room temperature (500 g), and the pellet was frozen at −80 °C for downstream processing. Remaining cultures (3 technical replicates, each with or without Shld1) were supplemented with E64 (15 µM) for further culture until 55 hpi for collection as described above.

Further protein extraction steps for each of the ten samples were carried out in solutions supplemented with cOmplete protease inhibitors (Roche) and PhosSTOP phosphatase inhibitor cocktail (Roche). We released parasites with 0.05% saponin in ice-cold PBS administered over several washes at 4 °C, for a total of ~6.5 volumes of buffer for 1 packed erythrocyte volume (PEV) of frozen pellet. Following additional washes in ice-cold PBS without saponin, we added >1 original PEV of 8 M urea lysis buffer (100 mM NaCl, 25 mM Tris-HCl/pH 8), and subjected each sample to 5× freeze (−80 °C)-thaw cycles before centrifugation at room temperature to separate protein-containing supernatant from pelleted cellular debris. Yields for each of the 10 samples ranged from 5-7 mg as assessed with the Pierce BCA (Bicinchoninic acid) protein assay. We reduced disulfide bonds with 5 mM tris(2-chloroethyl) phosphate (TCEP) for 30 min at room temperature, alkylated cysteines with 15 mM iodoacetamide for 30 min at room temperature in the dark, and quenched excess iodoacetamide by treatment with 5 mM dithiothreitol (DTT) for 15 min at room temperature. We precipitated protein in chloroform-methanol^86^ before resuspension (8 M urea, 50 mM HEPES pH 8.5) and before dilution of urea to 1 M (50 mM HEPES pH 8.5) for digestion with LysC protease (1:100 protease-to-protein ratio, 3 h at 37 °C) before the addition of trypsin (1:100 protease-to-protein ratio) and continued digestion overnight at 37 °C. We quenched the reaction with 1% formic acid, carried out C18 solid-phase extraction (Sep-Pak, Waters), and precipitated peptides with vacuum centrifugation.

To perform the isobaric labeling with tandem mass tags (TMTs), 200 µg of peptides from each sample was dissolved in Buffer 1 (100 mM HEPES, pH 8.5). We carried out labeling with TMT reagents^87^, according to manufacturer instructions (Thermo-Fisher Scientific). Following combination of the 10 TMT-labeled samples with matched protein mass between samples, the mixture was vacuum-centrifuged and subjected to C18 solid-phase extraction (Sep-Pak, Waters) and eluate was collected.

Peptides were resuspended in Buffer 1, followed by enrichment of phosphopeptides with High-Select™ Fe-NTA Phosphopeptide Enrichment Kit (Thermo-Scientific Cat. No. A32992)^88^. The flow-through was retained for analysis of the proteome. Peptides and enriched phosphopeptides were dried by vacuum centrifugation.

For proteomic analysis, the TMT-labeled peptide pool was fractionated via basic pH reversed-phase (BPRP) high-performance liquid chromatography^87^. Eluted fractions were desalted, dried by vacuum centrifugation, and resuspended in a solution of 5% acetonitrile and 5% formic acid, for mass spectrometry-based measurements.

For phosphopeptide analysis, we used the Pierce Off-line BPRP fractionation kit (Thermo Scientific), collecting and processing fractions for LC-MS/MS-based analysis^85^.

We collected MS/MS data using an Orbitrap Fusion mass spectrometer (Thermo Fisher Scientific) coupled to a Proxeon EASY-nLC 1000 liquid chromatography (LC) pump (Thermo Fisher Scientific). For each analysis, 1 µg protein was loaded onto the LC onto an in-house pulled C18 column [30–35 cm, 2.6 um Accucore (Thermo Fisher), 100 um ID] for MS-analysis^85^.

Global proteome and phosphoproteome analyses each employed the multi-notch MS3-based method^85,89^. Global proteome and phosphoproteome analyses used an MS3-based TMT method^90,91^, which has been shown to reduce ion interference compared to MS2 quantification^92^.

Mass spectra were analyzed with a SEQUEST-based pipeline^85,93^. Peptide spectral matches (PSMs) were carried out a 1% FDR, and filtered^85^. To quantify the TMT-reporter ion, in each channel (0.003 Th range to distinguish reporters) we extracted the summed signal-to-noise ratio and found the closest matching centroid to the expected mass. For proteomic analysis, PSMs (1% FDR) were collapsed to whole proteins (1% FDR). We used principles of parsimony to assemble the protein set, aiming to identify the smallest number of distinct polypeptides required to explain the observed PSMs. Relative protein levels were quantified by calculating the sum of reporter ion counts across associated PSMs^93^. MS3 spectra represented in <2 TMT channels, and MS3 spectra with TMT reporter summed spectra of <100, or no MS3 spectra, were not considered for further analysis^89^. Protein quantitation values were exported to Microsoft Excel. To normalize for variations in sample loading, within each TMT reporter ion channel, each parasite protein was normalized to the total signal of the *P. falciparum* proteome measured in that channel^85^. For phosphoproteomics, the signal intensity of each phosphopeptide in a single TMT channel was normalized to the level of the parent protein in the same experimental condition (average of normalized values for technical replicate measurements from proteomics). We did not apply this adjustment for the <2% of phosphopeptides that could not be mapped to a parent protein in proteomics. Raw signal intensities for non-phosphorylated peptides detected in phosphopeptide-enriched samples are reported in the Source Data (sheet: non-phos peptides), with column headings for the table as in Supplementary Data 3. For analysis in Supplementary Fig. 5d, non-phosphorylated peptide levels were normalized according to parent protein levels as described above for phosphopeptides.

For the 19 proteins shown to be increased in expression (>1.3-fold) in two proteomics measurements from separate experiments (Supplementary Data 1 and 2), we carried out gene ontology enrichment analysis using the webtool at PlasmoDB (http://www.plasmodb.org). The ten most enriched GO categories are shown in Supplementary Table 1.

For restriction of candidate substrates of *Pf*PP1 to proteins expressed late in the IDC, we used web-based tools at PlasmoDB (https://www.plasmodb.org) relying on published transcriptome data^16^ to identify *P. falciparum* genes that are upregulated at the 40- or 48 hpi-mark of the IDC by at least threefold over levels at the midpoint of the IDC (average expression of 16 and 24 hpi), and by at least 1.5-fold over levels at 32 hpi.

### Chemical-genetic assays

Compounds in DMSO (0.1–10 mM) were printed onto the surface of standard 96-well plates using a D300e automated dispenser (Hewlett Packard), and stored at −20 °C until addition of parasites. Chemicals in water were added individually onto the surface of 96-well plates, or added directly at the high concentration to parasites in wells before serial dilution in-plate. Phospholipids (or methanol vehicle) at 10x final concentration were added to the surface of plates and allowed to evaporate before addition of parasites. Synchronized *Pf*PP1-DD parasites were washed extensively in media without Shld1 and supplemented with varying concentrations of Shld1 (constant volume of ethanol carrier across doses), as indicated in the data. Parasites with Shld1, set at 0.5%-hematocrit, were added at 100 µl volume to wells with compounds to attain the reported final concentrations of inhibitors. Wells immediately surrounding the samples were filled with water or aqueous solution to prevent evaporation through the course of the assay. Parasites were allowed to incubate with inhibitor for ~20 h before fixation and measurement of egress from schizonts and reinvasion to rings by flow-cytometry.

For measurements in Supplementary Fig. 5a, 1 µl of calyculin (0.1 mM) was diluted to a final concentration of 500 nM directly into *Pf*PP1-DD parasites (0.3% hematocrit) with or without 0.5 µM Shld1 before manual serial dilution (fivefold) in 96-well plates.

### Reporting summary

Further information on research design is available in the Nature Research Reporting Summary linked to this article.

## Supplementary information

Supplementary Information

Reporting Summary

Description of Additional Supplementary Files

Supplementary Data 1

Supplementary Data 2

Supplementary Data 3

## Data Availability

The mass spectrometry proteomics data have been deposited to the ProteomeXchange Consortium (http://proteomecentral.proteomexchange.org) via the PRIDE partner repository^94^ with the dataset identifier PXD018718 and DOI 10.6019/PXD018718. All data generated or analyzed during this study are included in this published article (and its supplementary information files). A reporting summary for this article is available as a Supplementary Information file. Source data are provided with this paper.

## Source data

Source Data
