# Supplementary material for: Co-option of Plasmodium falciparum PP1 for egress from host erythrocytes
Source: Nat Commun. 2020 Jul 15;11:3532. doi: 10.1038/s41467-020-17306-1 (PMC7363832; doi:10.1038/s41467-020-17306-1)
Supplement: Supplementary file 1 — Supplementary Information [file 41467_2020_17306_MOESM1_ESM.pdf]

# Supplementary Information

Co-option of *Plasmodium falciparum* PP1 for egress from host erythrocytes

Paul *et al.*

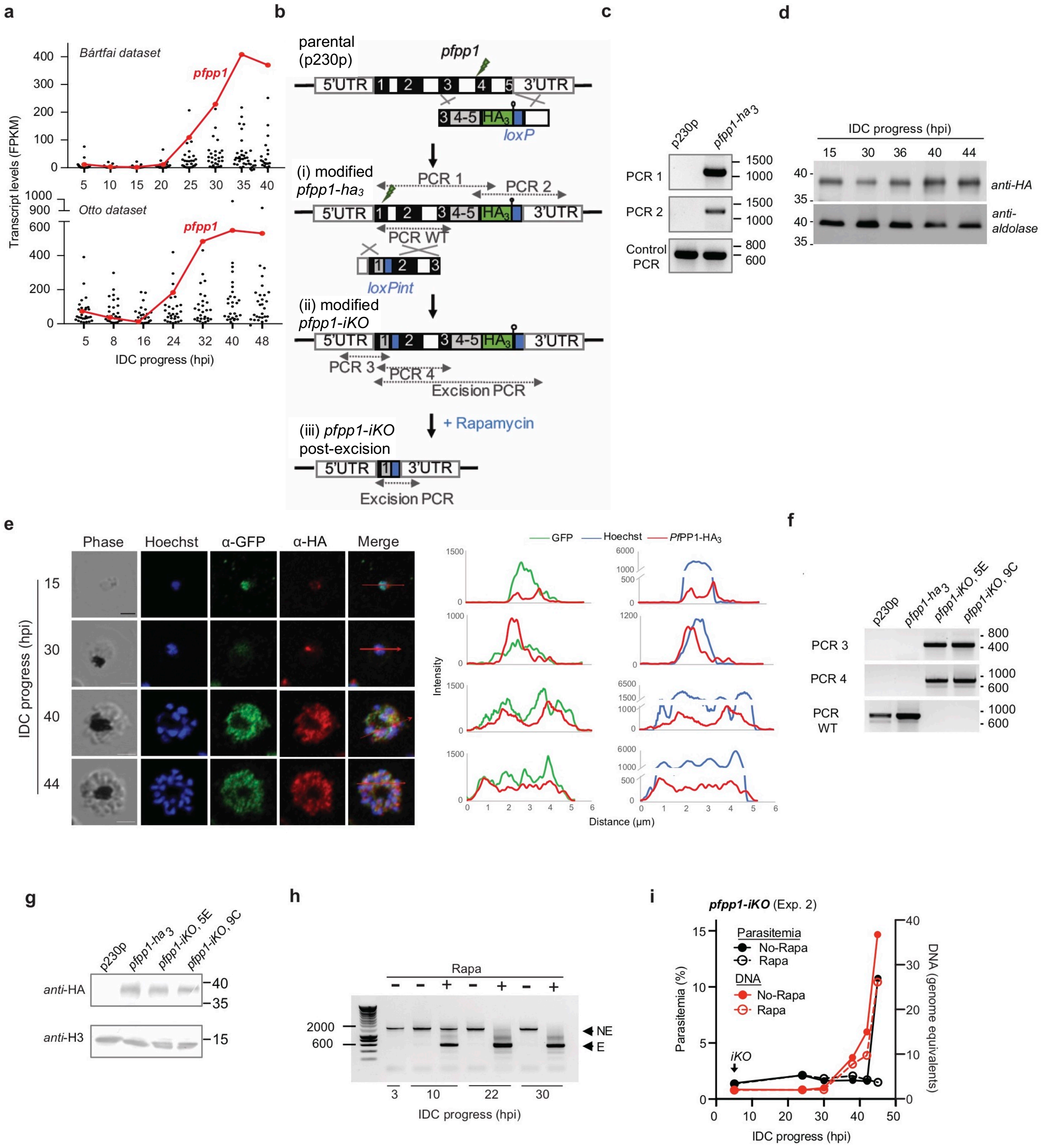


**Supplementary Figure 1.** *Genotypic validation and phenotypic assessment of pfpp1-ha₃ and pfpp1-iKO parasite lines.* **a** For 29 *P. falciparum* protein phosphatase genes[1], levels of mRNA expression (frequency per kilobase per millions reads, fpkm) over the course of the IDC measured in two separate transcriptome-wide studies from Bártfai and colleagues (top), and Otto and colleagues (bottom) [2,3]. **b** Sequential double-crossover recombination at the *pfpp1* chromosomal locus in the DiCre recombinase-expressing p230p parasite line to generate (i) the *pfpp1-ha$_3$* line with a

3’ loxP site; and (ii) the *pfpp1-iKO* with a loxP-containing synthetic intron (loxPint); followed by (iii) treatment with rapamycin to induce excision of *pfpp1*. Sites for PCR amplicons to confirm construction of transgenic lines are indicated. Black and white bars represent exons and introns of the *pfpp1* gene, respectively. Grey bars represent recodonized region of the *pfpp1* gene and blue bars stand for loxP site. Lollipop: stop codon; green lightning bolt: Cas9 double strand break. **c** PCRs for genotypic validation of *pfpp1-ha*$_3$ with 3’-loxP, as in b. Sizes are indicated in bp. Representative of 3 experiments. **d** Immunoblot of *Pf*PP1-HA$_3$ protein through the course of the IDC. *Pf*aldolase was used as a loading control. Molecular mass is indicated in kDa. Representative of 2 experiments. **e** Left: Localization of *Pf*PP1-HA$_3$ by immunofluorescence, relative to cytosolic GFP expressed from a pARL2-GFP episome and Hoechst-stained nuclei**.** Staging is indicated by IDC progress. Scale bar: 2 µm. Right: Profile of signal intensity of each channel along the line in the Merge images at left. Representative of 3 experiments. **f** PCRs for genotypic validation of *pfpp1-iKO* with loxP-containing synthetic intron, as in b. PCR also confirms the absence of unmodified *pfpp1* (PCR WT). Sizes are indicated in bp. Representative of 4 experiments. **g** Immunoblot performed with anti-HA antibodies to show that *pfpp1-ha*$_3$ and *pfpp1-iKO* parasite lines express a tagged version of the protein of the same molecular mass. Anti-histone H3 was used as a loading control (sample processing control on separate gel). Molecular mass is indicated in kDa. Representative of 1 experiment. **h** PCRs to confirm Rapa-induced excision of the *pfpp1* gene in *pfpp1-iKO* transgenic line following rapamycin treatment at 3 hpi. NE and E indicate the non-excised (1606 bp) and excised (581 bp) versions of *pfpp1* gene, respectively. Sizes are indicated in bp. Representative of 3 experiments. **i** A second experiment to measure the effect on DNA replication and proliferation in *pfpp1-iKO* parasites following +/- Rapa-treatment at 5 hpi, as in Fig. 1c. Mean of 3 technical replicates. Representative of 4 experiments. Source data are provided as a Source Data file.

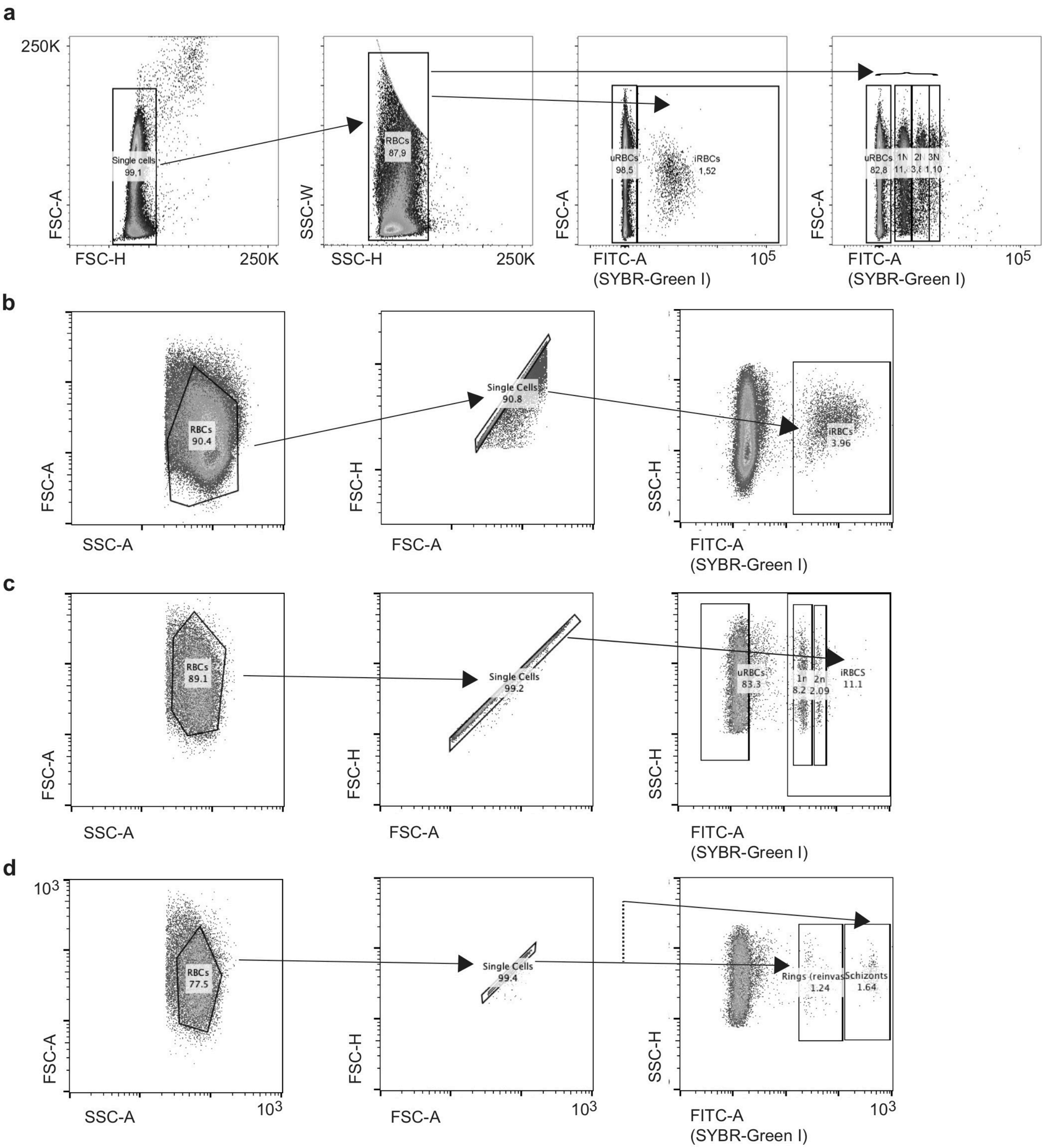


**Supplementary Figure 2.** *Gating strategies used for flow cytometry analysis.* **a** Gating strategy to calculate intraerythrocytic development of *pfpp1-iKO* infected erythrocytes (iRBCs), as in Figs. 1c and 2b, and Supplementary Figs. 1i and 4b. Linear scaling for all y-axes. Linear scaling for x-axes of two left two panels; $\log_{10}$ scaling for right two panels. **b** Gating strategy to measure proliferation of *Pf*PP1-DD-iRBCs observed in the iRBCs gate, as in Fig. 1e and Supplementary Fig. 3e. **c** Gating strategy to measure intraerythrocytic development of *Pf*PP1-DD parasites, based

on the mean fluorescent intensity of the iRBCs gate, as in Figs. 1f and 1h, and Supplementary Figs. 3g, 3h, 5a, and 5h. **d** Gating strategy to calculate (i) egress based on remaining cells in the Schizonts gate in endpoint samples as in Figs. 1h, 1j, 4d, and 4f; and Supplementary Figs. 3l, 3m, 5j, 6d, 6f, 6h, and 6i; or (ii) egress-to-invasion based on cells in the Rings gate in endpoint samples as in Figs. 1h, 1j, 3b, 3f, 3g, 4c, 4d, and 4g; and Supplementary Figs. 3l, 3m, 5i, 6b, 6c, 6e, and 6g. $Log_{10}$-scaling on both x- and y-axes in d applies for plots in same column above in b and c. In a and c, based on control samples with robust re-invasion to ring-stage parasites, DNA signal is calibrated according to uninfected RBCs (uRBCs), singly-infected rings (1N/n gate), and doubly-infected rings (2N/n gate), for 0, 1, and 2 genome equivalents, respectively. For *pfpp1-iKO* parasites, triply-infected parasites (3N gate) were included in the calibration.

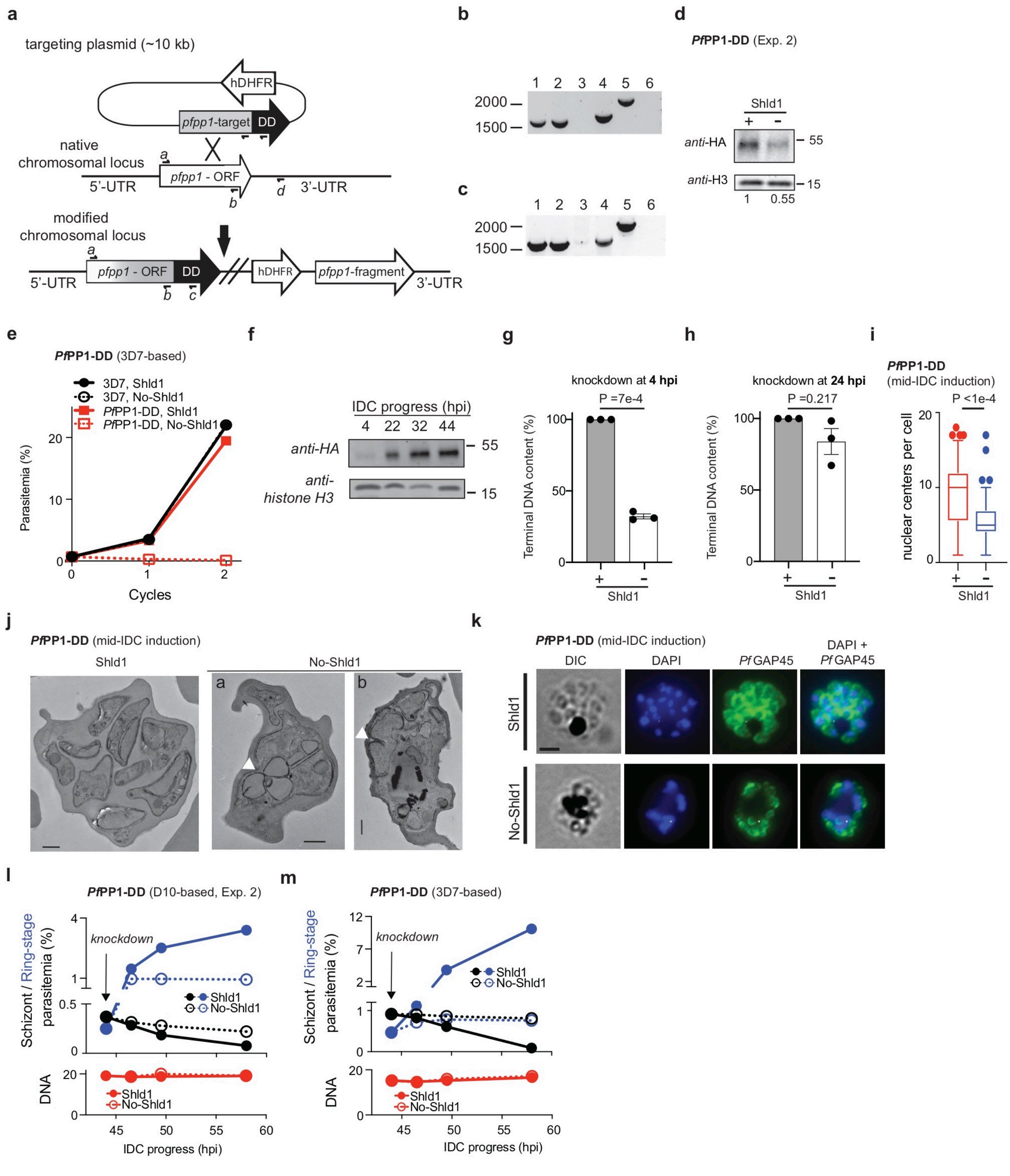


**Supplementary Figure 3.** *Genotypic validation and phenotypic assessment of PfPP1-DD parasites.* **a** Top: Scheme for single-crossover integration of the *dd-tag* into the endogenous *pfpp1* genetic locus, with binding sites for diagnostic primers indicated. **b-c** PCRs to confirm integration of *dd* into *pfpp1* in the D10 (b) and 3D7 background strains (c). Lanes 1,3,5: wild type parasite background; lanes 2,4,6: clonal *Pf*PP1-DD transgenic parasites. Lanes 1,2: diagnostic

primers *a,b*; lanes 3,4: primers *a,c*; lanes 5,6: primers *a,d*. Size in bp are indicated. Representative of 1 experiment per transgenic line. **d** An immunoblot of schizont-stage *Pf*PP1-DD parasites grown +/-0.2 μM Shld1 for ~20 hrs. Molecular mass is indicated in kDa. Representative of 1 experiment. **e** Proliferation of *Pf*PP1-DD in the 3D7 background, assessed as in Fig. 1e. Mean of 3 technical replicates. Representative of 2 experiments. **f** *Pf*PP1-DD expression in synchronized parasites in 0.2 μM Shld1 at the indicated timepoints following erythrocyte invasion, assessed with anti-HA tag antibody. Histone H3 levels were measured for loading control. Molecular mass is indicated in kDa. Representative of 1 experiment. **g-h** DNA content in advanced-stage parasites (51-55 hpi) +/- induction of *Pf*PP1-DD knockdown early (4 hpi, g) or at a mid-stage of the IDC (22-27 hpi, h), assessed by flow-cytometry. For both plots, mean +/- s.e.m.; n =3 independent experiments; two-tailed t test. **i** Box plot of numbers of nuclear centers (median with interquartile range; outliers at >95% percentile with none observed below 5%) in terminally developed parasites with induction of *Pf*PP1-DD knockdown at a mid-stage of the IDC (experiment from Fig. 1f, middle panel). +Shld1: n = 113 cells; mean, 9.0; min, 1; max 24. No-Shld1: n = 104 cells; mean, 5.5; min, 1; max, 17. Two-tailed Mann-Whitney test. Representative of 4 experiments. **j** Electron microscopy images of terminally developed *Pf*PP1-DD parasites (55 hpi), with knockdown induced at 24 hpi (+/-0.3 μM Shld1). Parasites were supplemented with 50 μM E64 at ~47 hpi to block egress. For knockdown-cells (No-Shld1), we show parasites arrested at a late stage when incipient cytokinesis in the vicinity of dividing nuclei are apparent (white arrowheads). Representative of 1 experiment. Scale bar: 0.5 μm. **k** Immunofluorescence of terminally developed *Pf*PP1-DD parasites, with knockdown and sampling as in j, indicating the inner membrane complex marker *Pf*GAP45. Scale bar: 2 μm. Representative of 2 experiments. **l-m** Egress and erythrocyte re-invasion following knockdown of *Pf*PP1-DD in either the D10 (l) or the 3D7 background (m) at 44 hpi, and DNA content in E64-treated parasites (50 μM) as described in Fig. 1h. Mean of 2 technical replicates for each panel. Representative of 2 (l) or 1 experiment (m). Source data are provided as a Source Data file.

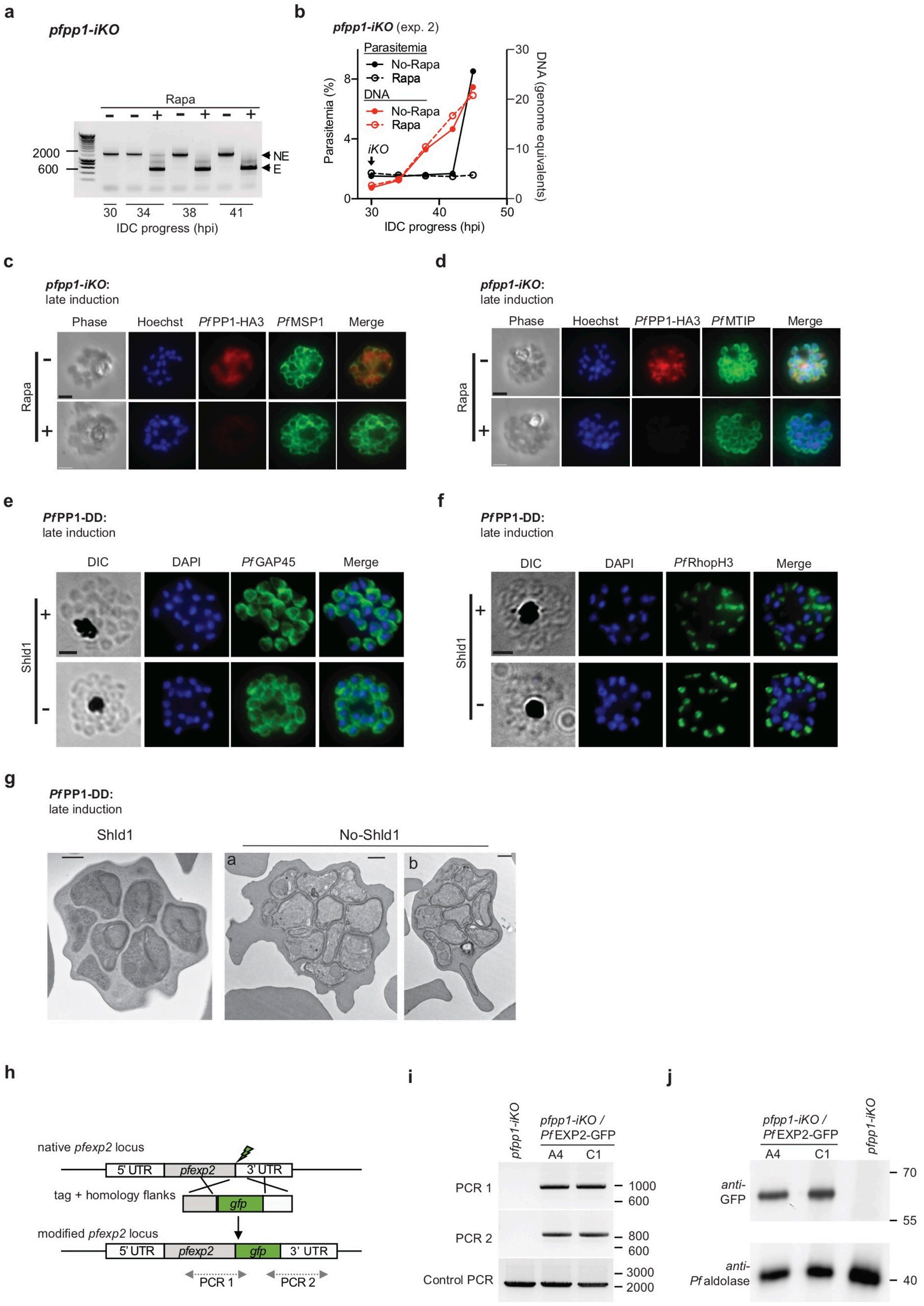
a
pfpp1-iKO
Rapa
- - + - + - +
2000
600
NE
E
30 34 38 41
IDC progress (hpi)
b
pfpp1-iKO (exp. 2)
Parasitemia
No-Rapa
Rapa
DNA
No-Rapa
Rapa
iKO
Parasitemia (%)
DNA (genome equivalents)
IDC progress (hpi)
c
pfpp1-iKO:
late induction
Phase
Hoechst
PfPP1-HA3
PfMSP1
Merge
Rapa
d
pfpp1-iKO:
late induction
Phase
Hoechst
PfPP1-HA3
PfMTIP
Merge
Rapa
e
PfPP1-DD:
late induction
DIC
DAPI
PfGAP45
Merge
Shld1
f
PfPP1-DD:
late induction
DIC
DAPI
PfRhopH3
Merge
Shld1
g
PfPP1-DD:
late induction
Shld1
No-Shld1
a
b
h
native pfexp2 locus
5' UTR
pfexp2
3' UTR
tag + homology flanks
gfp
modified pfexp2 locus
5' UTR
pfexp2
gfp
3' UTR
PCR 1
PCR 2
i
pfpp1-iKO
pfpp1-iKO / PfEXP2-GFP
A4
C1
PCR 1
PCR 2
Control PCR
1000
600
800
600
3000
2000
j
pfpp1-iKO / PfEXP2-GFP
A4
C1
pfpp1-iKO
anti-GFP
anti-Pfaldolase
70
55
40

**Supplementary Figure 4.** *Phenotypic assessment of late induction of pfpp1-iKO.* **a** PCRs to confirm excision of the *pfpp1* gene following iKO with rapamycin at 30hpi (trophozoite stage), as in Supplementary Fig. 1h. Sizes are indicated in bp. Representative of 3 experiments. **b** A second experiment measuring parasitemia and DNA synthesis following iKO of *pfpp1* at 30 hpi, as in Fig. 2b. Mean of 3 technical replicates. Representative of 4 experiments. **c-d** Immunofluorescence images showing depletion of *Pf*PP1-$HA_3$ in parasites assessed in Figs. 2e (c) and 2f (d). Scale bar: 2 µm. Each panel representative of 6 experiments. **e-f** Immunofluorescence microscopy images of *Pf*PP1-DD parasite cells, sampled at 55 hpi, indicating the inner membrane complex marker *Pf*GAP45 (e) or the rhoptry marker *Pf*RhopH3 (f), with knockdown (+/- 0.5 µM Shld1) induced at 44 hpi. Parasites were supplemented with 50 µM E64 to block egress. Scale bar: 2 µm. Representative of 2 experiments (e) or 1 experiment (f). **g** Electron microscopy images of terminally developed *Pf*PP1-DD parasites treated as in e-f. E64 was not used in No-Shld1 samples. Representative of 1 experiment. Scale bar: 0.5 µm. **h** Modification of the native *pfexp2* locus for 3'-tagging with GFP in the *pfpp1-iKO* background, via Cas9-mediated double-stranded break repair (see Supplementary Fig. 1b). **i** PCRs to confirm construction of *pfpp1-iKO / Pf*EXP2-GFP, as in h. Sizes are indicated in bp. Representative of 2 experiments. **j** Immunoblot to show PfEXP2-GFP expression in *pfpp1-iKO / Pf*EXP2-GFP lines. *Pf*aldolase was assessed to control for loading. Molecular mass is indicated in kDa. Representative of 1 experiment. Source data are provided as a Source Data file.

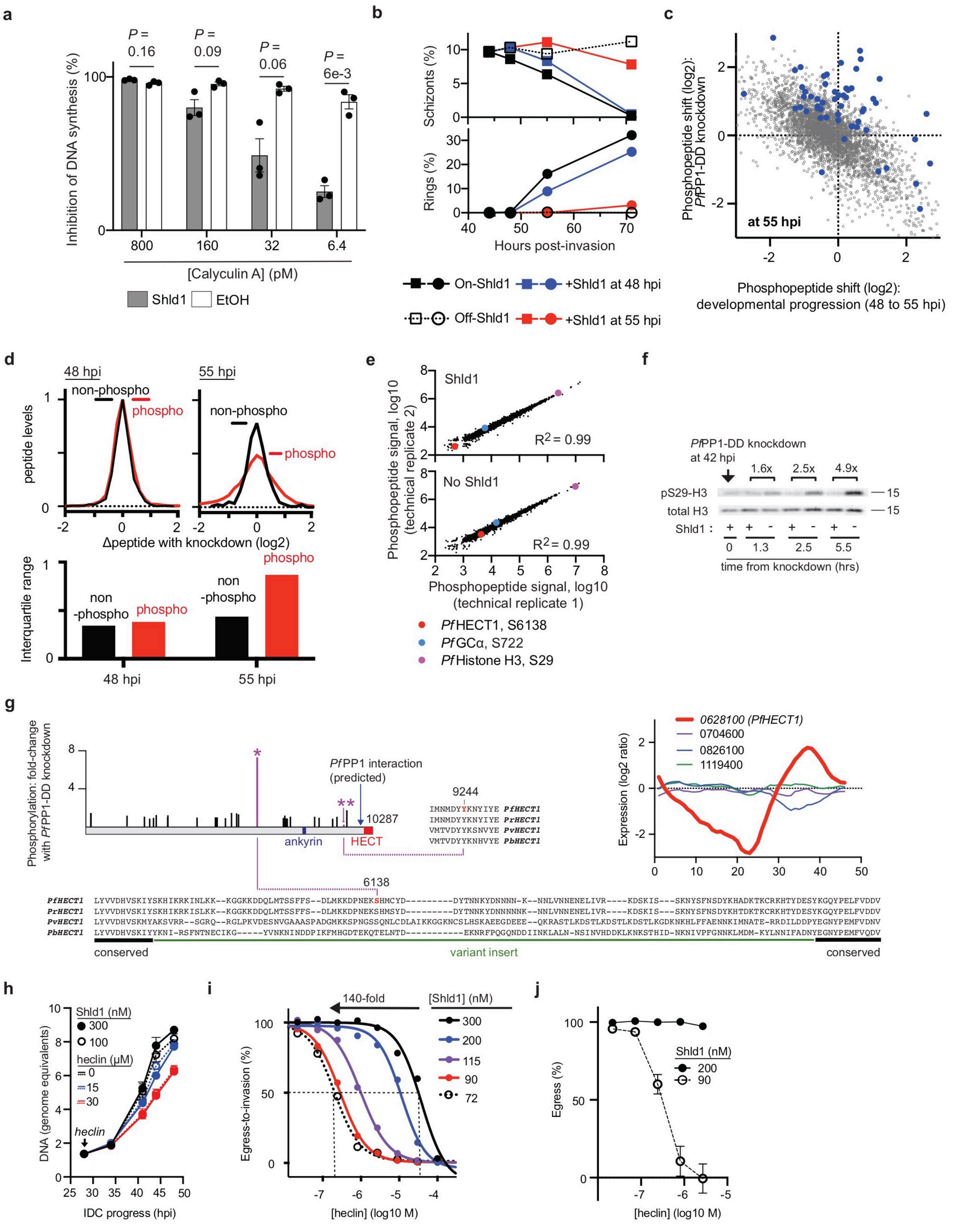


**Supplementary Figure 5.** *Phosphoproteomic analysis of PfPP1-DD knockdown and chemical-genetic analysis of*

*PfHECT1-mediated ubiquitination.* **a** Functional interaction of *Pf*PP1-DD knockdown with inhibition by calyculin A during parasite development. At 6 hpi (+/-4 hrs), we split parasites +/- 0.5 µM Shld1 and administered inhibitor at indicated concentrations before measurement of DNA content by flow-cytometry at 47 hpi. In each experiment, all data are normalized to measurements for inhibition in high calyculin A (0.5 µM, 100%) or DMSO vehicle (zero). Mean +/- s.e.m.; n=3 experiments; two-tailed t test. **b** Timecourse of schizont and ring-stage parasitemia in control samples for phosphoproteomic analysis of *Pf*PP1-DD knockdown, assessed from thin blood smears. Parasites were maintained continuously on-Shld1 (0.3 µM) or off-Shld1 (EtOH) following start of experiment at 44 hpi. To assess reversibility of the knockdown-phenotype, off-Shld1 parasites were supplemented with Shld1 (1 µM) at the two timepoints for sample collection. >49 infected cells counted per condition per timepoint. Representative of 1 experiment carried out in parallel with phosphoproteomics sample collection. **c** The effect of *Pf*PP1-DD knockdown on levels of all detected phosphopeptides at 55-hpi plotted against changes with development (+Shld1-parasites) from 48 to 55-hpi, as in Fig. 3d. All phosphopeptides upregulated by >2-fold at 48-hpi by *Pf*PP1-DD knockdown (Fig. 3d) are indicated in blue, showing that these hits are masked by developmental effects dominant at 55-hpi. **d** Top: The distribution of the log2-fold differences with *Pf*PP1-DD knockdown for either the enriched phosphopeptides (red) or non-phosphorylated peptides (black) measured in phosphopeptide-enriched samples, collected at the 48 (left) and 55-hpi timepoints (right). Bottom: The interquartile ranges for data shown in top panel. **e** In both the Shld1-supplemented and No Shld1 experimental conditions, phosphopeptide intensities at the 48-hpi timepoint. Each technical replicate is plotted against the other, with indication of squared correlation coefficient ($R^2$) of linear regression and *Pf*PP1-regulated phosphopeptides highlighted in Fig. 3d. **f** Phospho-*Pf*Histone H3 (Ser-29) dynamics following *Pf*PP1-knockdown in parasites at 42 hpi, assessed by immunoblot analysis. The fold-increase in phospho-*Pf*Histone H3 (S29), normalized to total *Pf*Histone H3 (sample processing control on separate gel), is indicated above the blot. Mean of 2 technical replicate timecourses. Molecular mass indicated in kDa. Representative of 1 experiment. **g** Left: Schematic of *Pf*HECT1 as in Fig. 3e with *Pf*PP1-regulated phosphosites shown in sequence alignment with orthologs from other *Plasmodium* spp. (*P. reichenowi*, *Pr; P. vivax*, *Pv; P. berghei, Pb*). For Ser-6138, extended sequence is shown to highlight regions of conservation and variation. Position of the predicted interaction site for *Pf*PP1 [4] is shown (blue arrow). Right: Relative transcript expression over the course of IDC [5] for the 4 HECT domain-containing proteins in *P. falciparum*, each denoted by the 7-digit suffix of the systematic gene ID beginning with "PF3D7_". **h** DNA replication in *Pf*PP1-DD parasites following administration of heclin at 28-hpi with indicated concentrations (DMSO

control, black; 15 μM, blue; 30 μM, red), and growth supported by a high (300 nM) or suboptimal (100 nM) concentration of Shld1. Mean +/- s.e.m.; n=3 experiments. **i** Dose-response curves from a single experiment to measure susceptibility of egress-to-invasion by *Pf*PP1-DD parasites to heclin at various Shld1 concentrations, with fold-change in IC50 values indicated. Mean of 2 technical replicates. Representative of 4 experiments. **j** The susceptibility of egress by *Pf*PP1-DD parasites to heclin at 200 or 90 nM Shld1. Mean +/- s.e.m.; n=3 experiments. Source data are provided as a Source Data file.

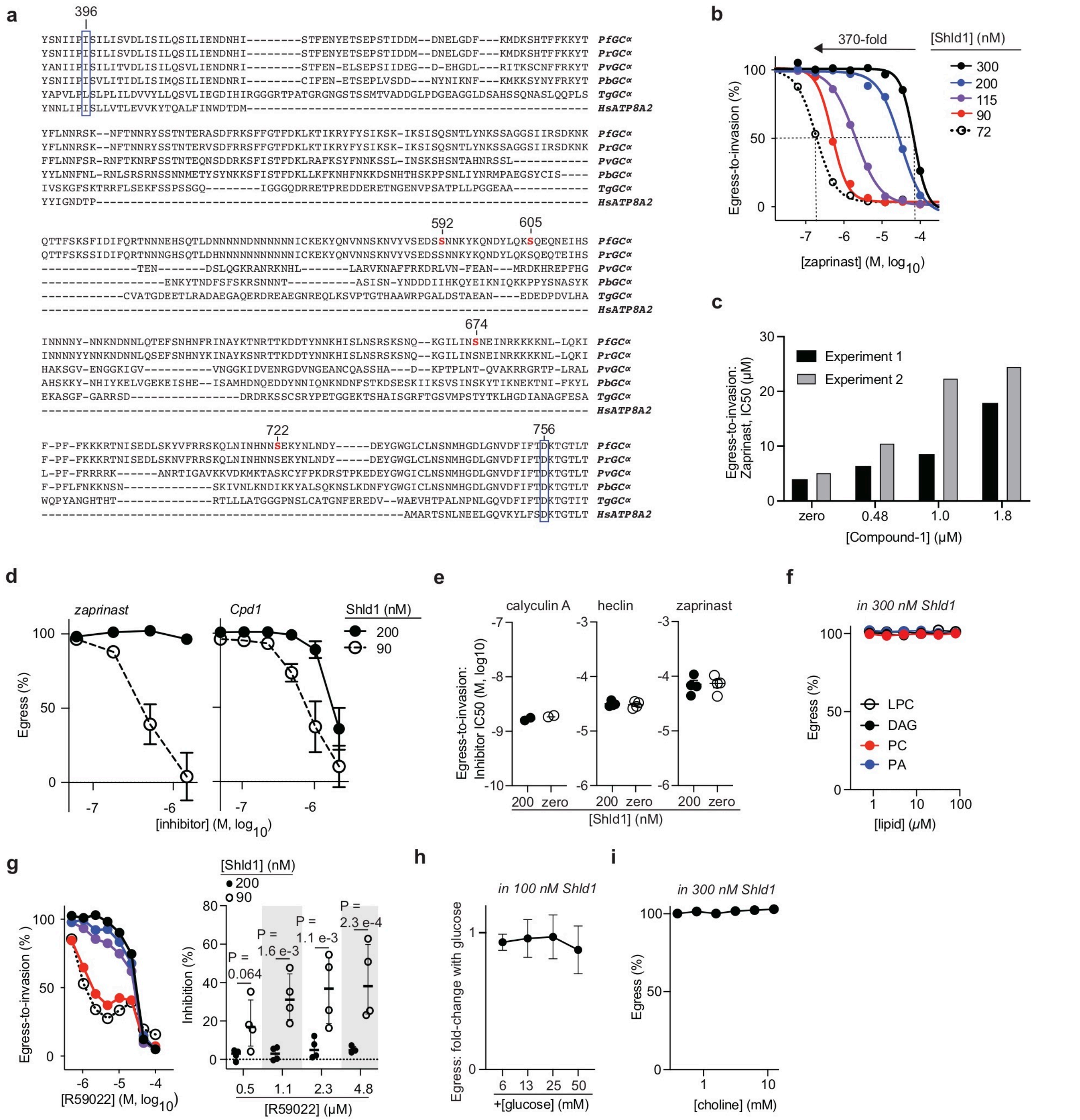


**Supplementary Figure 6.** *Chemical-genetic analysis of PfPP1-mediated signaling for egress.* **a** Alignment of the multi-phosphorylated cytoplasmic domain between the fourth and fifth transmembrane domains of the PLT in *Pf*GCα (see Fig. 4a). The alignment shows orthologs from *P. reichenowi* (*Pr*), *P. vivax* (*Pv*), *P. berghei* (*Pb*), and *T. gondii* (*Tg*); and also the human protein ATP8A2 (UniProt Q9NTI2.2) to indicate conservation of a isoleucine residue (396 in *Pf*GCα) in transmembrane 4 required for transport of phospholipids [6], indicated in purple. *Pf*PP1-responsive phosphorylation sites are indicated in red. The absolutely conserved catalytic aspartate residue in the consensus

sequence DKTGTLT required for ATPase-dependent phospholipid transport is indicated in purple. **b** An experiment with dose-responses of *Pf*PP1-DD parasites to zaprinast (egress-to-invasion) elicited over multiple Shld1 concentrations, with fold-change in IC50 values indicated. Mean of 2 technical replicates. Representative of 4 experiments. **c** Sensitivity to zaprinast of egress-to-invasion (IC50) of *Pf*PP1-DD parasites in 115 nM Shld1 and fixed concentrations of Compound-1 as indicated, measured as in Fig. 4c. The results of 2 independent experiments are shown. **d** Sensitivity of egress by *Pf*PP1-DD parasites to zaprinast (left) or Cpd1 (right). Mean +/- s.e.m.; n=3 experiments. **e** The sensitivity of egress-to-invasion by D10 parasites (parent to *Pf*PP1-DD) for the indicated compounds at the indicated Shld1 concentrations. Calyculin: mean; n = 2 experiments. Heclin and zaprinast: mean +/- s.e.m.; n=4 independent experiments. **f** In 300 nM Shld1, percent egress for *Pf*PP1-DD parasites in the presence of the lipids in Fig. 4d at the indicated concentrations. Mean +/- s.e.m.; DAG and PA: n=3 experiments; LPC: n=4 experiments; PC: n=7 experiments. **g** Left: A series of dose-response curves in a single experiment showing induced sensitivity of parasite egress-to-invasion to R59022 with knockdown of *Pf*PP1-DD. Shld1 concentrations as in b. Mean of 2 technical replicates. Representative of 4 experiments. Right: Induced sensitivity of parasites to low concentrations (<10 µM) of R59022 with partial knockdown of *Pf*PP1-DD, based on inhibition of egress-to-invasion. Mean +/- s.e.m.; n=4 experiments; multiple two-tailed t tests (false discovery rate, 1%). **h** In 100 nM Shld1, the effect of supplementation with the indicated concentrations of glucose on egress by *Pf*PP1-DD parasites, expressed as fold-change compared to no-additional glucose. Mean +/- s.e.m.; n=4 experiments. The baseline concentration of glucose in media is 11.1 mM. **i** In 300 nM Shld1, percent egress for choline (see Fig. 4f) at indicated concentrations, as in f. Mean +/- s.e.m.; n=4 experiments. Source data are provided as a Source Data file.

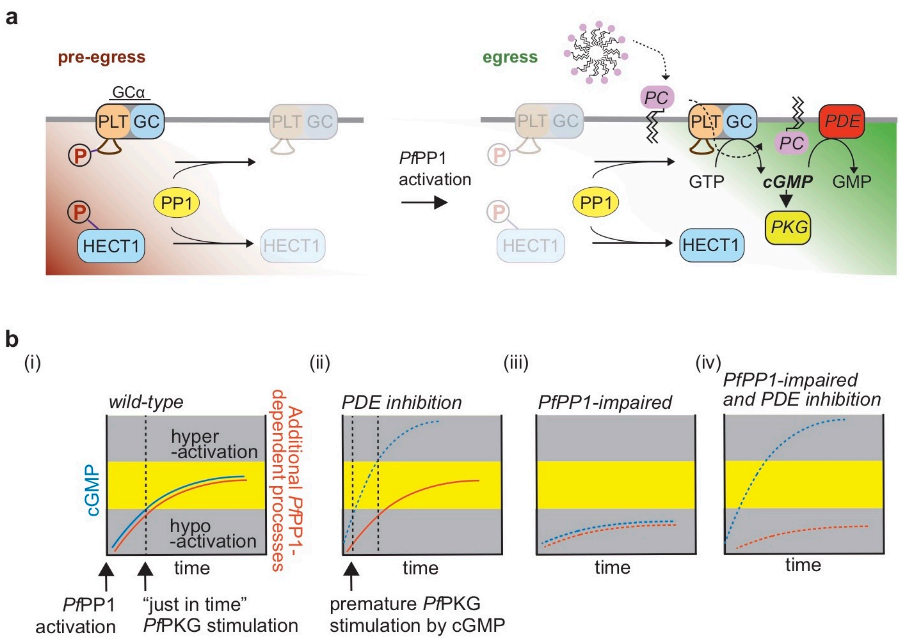


**Supplementary Figure 7.** *A model for coordination by PfPP1 of cell-intrinsic pathways and environmental signals for egress.* **a** Before activation of *Pf*PP1, the parasite is in a pre-egress state where substrates of the protein phosphatase are phosphorylated. With *Pf*PP1 activation, the parasite enters the egress state by dephosphorylating *Pf*HECT1 and *Pf*GCα. We propose that dephosphorylation of the PLT domain directly regulates cGMP synthesis at the GC domain, and/or permits translocation of extra-parasitic PC to initiate cGMP synthesis and stimulate *Pf*PKG. **b** Just in time stimulation of *Pf*PKG and additional functions dependent on *Pf*PP1, with (i) coordinated activation in wild-type parasites (yellow band) resulting in egress. (ii)-(iv) depict how PDE inhibition and/or *Pf*PP1 disruption of function may result in failure of parasites to egress. (ii) Inhibition of PDE hyperactivates cGMP synthesis for premature stimulation of *Pf*PKG prior to additional processes. (iii) *Pf*PP1 impairment reduces activation of all processes dependent on the protein phosphatase. (iv) *Pf*PP1 impairment aggravates PDE inhibition-induced miscoordination of *Pf*PKG function and additional processes for egress.

**Supplementary Table 1.** *Gene Ontology analysis of proteins upregulated with PfPP1-DD knockdown in late IDC-stage parasites*

| ID[a] | Name[b] | Bgd count[c] | Result count[d] | Result gene list[e] | P-value[f] | Benjamini[g] | Bonferroni[h] |
|---|---|---|---|---|---|---|---|
| GO:0006098 | pentose-phosphate shunt | 9 | 3 | PF3D7_1436000, PF3D7_1439900, PF3D7_1454700 | 8.78e-6 | 8.34e-4 | 2.50e-3 |
| GO:0006739 | NADP metabolic process | 9 | 3 | PF3D7_1436000, PF3D7_1439900, PF3D7_1454700 | 8.78e-6 | 8.34e-4 | 2.50e-3 |
| GO:0051156 | glucose 6-phosphate metabolic process | 9 | 3 | PF3D7_1436000, PF3D7_1439900, PF3D7_1454700 | 8.78e-6 | 8.34e-4 | 2.50e-3 |
| GO:0019682 | glyceraldehyde-3-phosphate metabolic process | 10 | 3 | PF3D7_1436000, PF3D7_1439900, PF3D7_1454700 | 1.25e-5 | 8.91e-4 | 3.56e-3 |
| GO:0006081 | cellular aldehyde metabolic process | 12 | 3 | PF3D7_1436000, PF3D7_1439900, PF3D7_1454700 | 2.28e-5 | 1.30e-3 | 6.49e-3 |
| GO:0019362 | pyridine nucleotide metabolic process | 21 | 3 | PF3D7_1436000, PF3D7_1439900, PF3D7_1454700 | 1.34e-4 | 5.25e-3 | 3.82e-2 |
| GO:0046496 | nicotinamide nucleotide metabolic process | 21 | 3 | PF3D7_1436000, PF3D7_1439900, PF3D7_1454700 | 1.34e-4 | 5.25e-3 | 3.82e-2 |
| GO:0072524 | pyridine-containing compound metabolic process | 24 | 3 | PF3D7_1436000, PF3D7_1439900, PF3D7_1454700 | 2.02e-4 | 5.25e-3 | 5.76e-2 |
| GO:0006733 | oxidoreduction coenzyme metabolic process | 25 | 3 | PF3D7_1436000, PF3D7_1439900, PF3D7_1454700 | 2.29e-4 | 5.25e-3 | 6.52e-2 |
| GO:0006006 | glucose metabolic process | 5 | 2 | PF3D7_1436000, PF3D7_1439900 | 2.39e-4 | 5.25e-3 | 6.82e-2 |

[a] 10 most significantly enriched functional categories by GO ID

[b] Descriptive name for GO category

[c] Total number of *P. falciparum* genes attributed to the GO category

[d] Number of proteins in category found upregulated

[e] Upregulated proteins by *P. falciparum* systemic gene ID

[f] For null hypothesis that GO category is not enriched

[g] Benjamini adjustment for multiple hypothesis testing

[h] Bonferroni adjustment for multiple hypothesis testing

**Supplementary Table 2.** *Primers used in this study*

| Primer | Sequence 5'-3' | Purpose |
|---|---|---|
| AK15 | gctcatgcggccgctagatatagataatgtaatatcaaa actaatagaag | Forward primer for amplification of *pfpp1* from genomic DNA |
| AK16 | gctcatctcgagatttgctgctttcttttttccactg | Reverse primer for amplification of *pfpp1* from genomic DNA; primer b in Supplementary Figure 3a |
| AP266 | ggaaggtgcgcccgtc | Primer c in Supplementary Figure 3a |
| AP400 | caagtaaatatttattatggcattag | Primer a in Supplementary Figure 3a |
| AP464 | ctacttttatagataatatttgttttgttc | Primer d in Supplementary Figure 3a |
| MLa118 | ctgcccggggattatgtggatagaggaaaacaaag | Forward primer for amplification of *pfpp1* from genomic DNA (HA3 tagging) |
| MLa119 | ctgatgtgccctgcatattaaatc | Reverse primer for amplification of *pfpp1* from genomic DNA (HA3 tagging) |
| MLa120 | atatgcagggcacatcagg | Forward primer for amplification of recodonized *pfpp1* from GBlock (HA3 tagging) |
| MLa121 | tgtagcgctattggccgctttcttttttc | Reverse primer for amplification of recodonized *pfpp1* from GBlock (HA3 tagging) |
| MLa178 | cgcctgcagtatattacaaaacttaggatcctaatatatta attg | Forward primer for amplification of *pfpp1* 3'UTR from genomic DNA |
| MLa179 | ctggttaacgaaaaatactacttttatagataatatttgtttt gttc | Reverse primer for amplification of *pfpp1* 3'UTR from genomic DNA |
| MLa206 | tattgtcaacactcatcattgcac | Forward gRNA *pfpp1* (HA3 tagging) |
| MLa207 | aaacgtgcaatgatgagtgttgac | Reverse gRNA *pfpp1* (HA3 tagging) |
| MLa264 | ctcaagcttggggggatccatatcaaataaaataaattc attcttc | Forward primer for amplification of *pfpp1* from genomic DNA (loxPint) |
| MLa265 | tggaaatccaccatattcaaataac | Reverse primer for amplification of *pfpp1* from genomic DNA (loxPint) |
| MLa266 | gtgattatgtggatagaggaaaacaaag | Forward primer for amplification of *pfpp1* from genomic DNA (loxPint) |
| MLa267 | gaattagctaagcatgcgctcgatcattttctccccacc | Reverse primer for amplification of *pfpp1* from genomic DNA (loxPint) |
| MLa268 | gaatatggtggatttccaccag | Forward primer for amplification of recodonized *pfpp1* from GBlock (loxPint) |
| MLa269 | ctctatccacataatcacctaaaag | Reverse primer for amplification of recodonized *pfpp1* from GBlock (loxPint) |
| MLa287 | tattaaaaatagataatttgcatc | Forward gRNA *pfpp1* (loxPint) |
| MLa288 | aaacgatgcaaattatctattttt | Reverse gRNA *pfpp1* (loxPint) |
| MLa187 | catggacagttttatgatttgttaagg | Forward primer for PCR1 in Supplementary Figure 1c |
| ML1476 | cagcgtagtccgggacgtcgtac | Reverse primer for PCR1 in Supplementary Figure 1c |
| MLa244 | agaaagcggccaatagctacccgtacgacgtcc | Forward primer for PCR2 in Supplementary Figure 1c |
| MLa188 | gaaatatatggctaaattaaatataaatagc | Reverse primer for PCR2 in Supplementary Figure 1c |
| MLa11 | cgcctcgagatgaagagtttggagaataacg | Forward primer for control PCR in Supplementary Figure 1c |
| MLa12 | cgcggtacccataaaatgacatttcctaagac | Reverse primer for control PCR in Supplementary Figure 1c |
| MLa296 | cataaaattaaaggacacataaagac | Forward primer for PCR3 in Supplementary Figure 1f |
| MLa307 | gaacaagtagttggcgtctgg | Reverse primer for PCR3 in Supplementary Figure 1f |
| MLa306 | ccagacgccaactacttgttc | Forward primer for PCR4 in Supplementary Figure 1f |
| MLa119 | ctgatgtgccctgcatattaaatc | Reverse primer for PCR4 and PCR WT in Supplementary Figure 1f |
| MLa311 | cgatgcaaattatctatttttagg | Forward primer for PCR WT in Supplementary Figure 1f |
| MLa336 | cgtgggcccgccttgagagaaatatgggat | Forward primer for amplification of *pfexp2* 3'coding sequence from genomic DNA |
| MLa337 | cgccgtacgttctttattttcatcttttttttcatttttaaataa atctccac | Reverse primer for amplification of *pfexp2* 3'coding sequence from genomic DNA |
| MLa338 | cgccttaagggagaaacaatcttttatataaaatgtacag agtttgaaag | Forward primer for amplification of *pfexp2* 3'UTR from genomic DNA |
| MLa339 | cgcctgcagtcataaggagagtacataaataaaataatc aaac | Reverse primer for amplification of *pfexp2* 3'UTR from genomic DNA |
| MLa340 | tattatattatgtacagtatctga | Forward gRNA *pfexp2* |
| MLa341 | aaactcagatactgtacataatat | Reverse gRNA *pfexp2* |
| MLa344 | gtattaagatggtcacgtatgtgg | Forward primer for PCR1 in Supplementary Figure 4i |
| MLa345 | ttgtgcccattaacatcacc | Reverse primer for PCR1 in Supplementary Figure 4i |
| MLa346 | atcccaacgaaaagagagacc | Forward primer for PCR2 in Supplementary Figure 4i |
| MLa347 | gttgtatagagacacaattcgtataacac | Reverse primer for PCR2 in Supplementary Figure 4i |
| MLa16 | cgcctcgagatggaaccactgcctgatcc | Forward primer for control PCR in Supplementary Figure 4i |
| MLa17 | cgcggtaccttcgttggatggcctcttttc | Reverse primer for control PCR in Supplementary Figure 4i |

**Supplementary Note 1.** *Specificity of PfPP1-function according to phosphoproteomic data.* At 48-hpi, 30 phosphopeptides (0.8% of 3884 total detected) exhibit a reduction of >2-fold with *Pf*PP1-DD destabilization, and 60 (1.5% of total) are increased by >2-fold (Supplementary Data file 3). Other evidence suggests limited effects secondary to perturbation of *Pf*PP1-function at 48-hpi: (i) proliferation defects elicited by knockdown are reversible to a large degree with resupplementation of Shld1 at 48-hpi, but not 55-hpi (Supplementary Fig. 5b); (ii) in contrast to 55-hpi, changes in the global phosphoproteome with *Pf*PP1-DD knockdown at 48-hpi are not predictive of further developmental progression (Fig. 3d; Supplementary Fig. 5c); and (iii) overall variability in the phosphopeptide levels at 48-hpi, but not 55-hpi, induced by *Pf*PP1-DD knockdown is comparable to a baseline, determined from the distribution of non-phosphorylated peptides[7] in the same sample (Supplementary Fig. 5d). Of the phosphopeptides upregulated by >2-fold with *Pf*PP1-DD knockdown at 48-hpi (y-axis, Fig. 3d), we regard sites that did not decrease with IDC progression from 48 to 55-hpi (x-axis, Fig. 3d) to be least likely to be influenced by effects secondary to perturbation of *Pf*PP1. The *Pf*PP1-regulated phosphopeptides for *Pf*HECT1, *Pf*GC$\alpha$, and *Pf*Histone H3, are well correlated between technical replicates (Supplementary Fig. 5e).

## Supplementary References


1. Guttery, D. S. *et al.* Genome-wide Functional Analysis of *Plasmodium* Protein Phosphatases Reveals Key Regulators of Parasite Development and Differentiation. *Cell Host & Microbe* **16,** 128–140 (2014).
2. Bártfai, R. *et al.* H2A.Z Demarcates Intergenic Regions of the *Plasmodium falciparum* Epigenome That Are Dynamically Marked by H3K9ac and H3K4me3. *PLoS Pathog* **6,** e1001223–14 (2010).
3. Otto, T. D. *et al.* New insights into the blood-stage transcriptome of *Plasmodium falciparum* using RNA-Seq. *Molecular Microbiology* **76,** 12–24 (2010).
4. Hollin, T., De Witte, C., Lenne, A., Pierrot, C. & Khalife, J. Analysis of the interactome of the Ser/Thr Protein Phosphatase type 1 in *Plasmodium falciparum*. *BMC Genomics* **17,** 246 (2016).
5. Bozdech, Z. *et al.* The transcriptome of the intraerythrocytic developmental cycle of *Plasmodium falciparum*. *PLoS Biol* **1,** E5 (2003).
6. Vestergaard, A. L. *et al.* Critical roles of isoleucine-364 and adjacent residues in a hydrophobic gate control of phospholipid transport by the mammalian P4-ATPase ATP8A2. *Proc. Natl. Acad. Sci. U.S.A.* **111,** E1334–43 (2014).
7. Invergo, B. M. *et al.* Sub-minute Phosphoregulation of Cell Cycle Systems during *Plasmodium* Gamete Formation. *Cell Reports* **21,** 2017–2029 (2017).
