# Supplementary material for: Co-option of Plasmodium falciparum PP1 for egress from host erythrocytes
Source: Nat Commun. 2020 Jul 15;11:3532. doi: 10.1038/s41467-020-17306-1 (PMC7363832; doi:10.1038/s41467-020-17306-1)
Supplement: Supplementary file 2 — Reporting Summary [file 41467_2020_17306_MOESM2_ESM.pdf]

nature research

Corresponding author(s): Manoj T. Duraisingh
Mauld H. Lamarque

Last updated by author(s): Jun 16, 2020

Reporting Summary

Nature Research wishes to improve the reproducibility of the work that we publish. This form provides structure for consistency and transparency in reporting. For further information on Nature Research policies, see Authors & Referees and the Editorial Policy Checklist .

Statistics

For all statistical analyses, confirm that the following items are present in the figure legend, table legend, main text, or Methods section.

n/a	Confirmed	
☐	☒	The exact sample size (n) for each experimental group/condition, given as a discrete number and unit of measurement
☐	☒	A statement on whether measurements were taken from distinct samples or whether the same sample was measured repeatedly
☐	☒	The statistical test(s) used AND whether they are one- or two-sided<br>*Only common tests should be described solely by name; describe more complex techniques in the Methods section.*
☒	☐	A description of all covariates tested
☐	☒	A description of any assumptions or corrections, such as tests of normality and adjustment for multiple comparisons
☐	☒	A full description of the statistical parameters including central tendency (e.g. means) or other basic estimates (e.g. regression coefficient) AND variation (e.g. standard deviation) or associated estimates of uncertainty (e.g. confidence intervals)
☐	☒	For null hypothesis testing, the test statistic (e.g. F, t, r) with confidence intervals, effect sizes, degrees of freedom and P value noted<br>*Give P values as exact values whenever suitable.*
☒	☐	For Bayesian analysis, information on the choice of priors and Markov chain Monte Carlo settings
☒	☐	For hierarchical and complex designs, identification of the appropriate level for tests and full reporting of outcomes
☒	☐	Estimates of effect sizes (e.g. Cohen's d, Pearson's r), indicating how they were calculated

Our web collection on statistics for biologists contains articles on many of the points above.

Software and code

Policy information about availability of computer code

Data collection	*Provide a description of all commercial, open source and custom code used to collect the data in this study, specifying the version used OR state that no software was used.*
Data analysis	For IFA images processing, ZEN 2 blue edition (v2.0.0.0 or v2.3.69.1000) was used. For data analysis, we used Prism version 8.4

For manuscripts utilizing custom algorithms or software that are central to the research but not yet described in published literature, software must be made available to editors/reviewers. We strongly encourage code deposition in a community repository (e.g. GitHub). See the Nature Research guidelines for submitting code & software for further information.

Data

Policy information about availability of data

All manuscripts must include a data availability statement. This statement should provide the following information, where applicable:

- Accession codes, unique identifiers, or web links for publicly available datasets
- A list of figures that have associated raw data
- A description of any restrictions on data availability

The authors declare that all the data supporting the findings of this study are available within the paper and its supplementary information files. The source data underlying Figures 1a-j, 2a-c, 2h-j, 3b, 3f-g, and 4b-g and Supplementary Figures 1c-d, 1f-i, 3b-i, 3l-m, 4a-b, 4i-j, 5a-b, 5f, 5h-j and 6b-i, are provided as a source data file. The mass spectrometry proteomics data have been deposited to the ProteomeXchange Consortium (http://proteomecentral.proteomexchange.org) via the PRIDE partner repository (94) with the dataset identifier PXD018718 and DOI 10.6019/PXD018718.

94. Vizcaíno, J. A. et al. 2016 update of the PRIDE database and its related tools. Nucleic Acids Research 44, D447–D456 (2016).

Field-specific reporting

Please select the one below that is the best fit for your research. If you are not sure, read the appropriate sections before making your selection.

- [x] Life sciences
- [ ] Behavioural & social sciences
- [ ] Ecological, evolutionary & environmental sciences

For a reference copy of the document with all sections, see nature.com/documents/nr-reporting-summary-flat.pdf

Life sciences study design

All studies must disclose on these points even when the disclosure is negative.

Sample size	For parasite nuclear counts, we counted at least 54 and up to 199 infected erythrocytes, similar to previous work [Reilly, H. B., et al (2007) International Journal for Parasitology. 37, 1599–1607].<br>For counts of parasitemia, by thin blood smears we counted at least 409 total erythrocytes similar to standards prescribed by the CDC (https://www.cdc.gov/dpdx/diagnosticprocedures/blood/microexam.html). The number of cells we counted by flow cytometry (>20000) far exceeds this minimum, providing even greater power to calculate parasitemia.<br>To determine PVM integrity and organelle secretion in PfPP1-iKO by IFA, at least 1500 cells were examined per condition (Mock or Rapamycin-treatment) over 3 independent experiments, similarly to previous studies (Collins R.C. et al. (2013) PLoS Pathogens. 9 (5): e1003344 ; Absalon S. et al. (2018) mBio 9:e00130-18).<br>To assess labeling of parasites with phosphatidylcholine, we counted a minimum of 40 cells per sample, similar to sample sizes reported for microscopy-based measurements of parasite behavior related to egress and invasion [Weiss et al (2015) PLoS Pathog. 11, e1004670–25].
Data exclusions	For the proteomic data, we implemented a minimum signal threshold for inclusion in downstream analyses previously implemented in Ganter et al (2017, Nature Microbiology. 2, 17017).
Replication	Exact values for replication number are provided in legends associated with data.<br>For PfPP1-iKO parasites and D10-based PfPP1-DD parasites, we measured parasite proliferation (1 cycle or multiple cycles) at least twice. We carried out 1 additional experiment for 3D7-based PfPP1-DD, providing a biological replicate.<br>All intraerythrocytic developmental studies (n=3-4)<br>Counts of nuclei per schizont (n=3-4)<br>PVM integrity and organelle secretion (n=3)<br>Proteomic analysis (n=2)<br>Phosphoproteomic analysis (n=1)<br>Chemical-genetic analysis for PfPP1-DD parasites (n=4); chemical-genetics for D10 wild-type parasites (n=2-4)
Randomization	Independent variables in this study are (i) genetic modifications, (ii) concentrations of chemical reagents, and (iii) time.<br>(i) All genetic modifications were validated through genotypic analysis; and conditional expression of proteins was validated through Immunoblot analysis.<br>(ii) Chemical doses were controlled with volumetric containers (e.g. serological pipettes) or mechanical pipettes calibrated on a regular basis.<br>(iii) We followed time using standard clocks.
Blinding	All the IFAs for PVM integrity, organelle secretion assays and nuclei numbers in pfpp1-iKO were quantified by blind measurements. Other analyses derived from instrument-based measurements were less prone to user bias, and we did not blind these.

Reporting for specific materials, systems and methods

We require information from authors about some types of materials, experimental systems and methods used in many studies. Here, indicate whether each material, system or method listed is relevant to your study. If you are not sure if a list item applies to your research, read the appropriate section before selecting a response.

Materials & experimental systems

n/a	Involved in the study	
☐	☒	Antibodies
☐	☒	Eukaryotic cell lines
☒	☐	Palaeontology
☒	☐	Animals and other organisms
☐	☒	Human research participants
☒	☐	Clinical data

Methods

n/a	Involved in the study	
☒	☐	ChIP-seq
☐	☒	Flow cytometry
☒	☐	MRI-based neuroimaging

Antibodies

Antibodies used	rabbit anti-histone H3 (Abcam ab1791); rat anti-HA antibody 3F10 (Roche Cat. No. 11867423001); anti-phospho S28 histone H3 antibody (Abcam Cat. No. ab5169); mouse anti-RhopH3 (gift from Jean-Francois Dubremetz); rabbit anti-PfGAP45 (gift from Dr. Julian Rayner, Wellcome Trust Sanger Institute, Hinxton, UK); rabbit anti-MTIP (gift from Tony Holder, The Francis Crick Institute, UK); mouse anti-MSP1.19 (gift from M. Blackman, The Francis Crick Institute, UK); mouse anti-RON4 (home made); mouse anti-SUB1 (gift from M. Blackman, The Francis Crick Institute, UK); rabbit anti-AMA1 (gift from M. Blackman, The Francis Crick Institute, UK); rabbit anti-Plasmodium-aldolase-HRP (Abcam ab38905); mouse anti-GFP (Roche, Cat. No. 11814460001).<br>For IFA studies, the secondary antibodies used were Alexa Fluor 488 and 594-conjugated antibodies against mouse, rat or rabbit IgG (Invitrogen A21208, A11012, A11001, A11008, A21209 and A11005).
Validation	- ab1791 is noted by the manufacturer to detect histone H3 protein in multiple eukaryotic species, including the parasite species Toxoplasma gondii, a member of the apicomplexan phylum alongside Plasmodium (https://www.abcam.com/histone-h3-antibody-nuclear-loading-control-and-chip-grade-ab1791.html). We and others have used the antibody in Plasmodium falciparum for immunoblotting analysis of parasite histone H3 (Flueck et al (2009) PLoS Pathogens 5: e1000569; Salcedo-Amaya (2009) Proc Nat Acad Sci 24: 9655; Paul et al (2015) Cell Host Microbe 18: 49)<br>- 3F10 is commonly used for anti-HA in P. falciparum. Validation is provided by detection of knockdown of HA-tagged proteins in this study and others (Paul, A. S., et al (2015); Cell Host & Microbe. 18, 49–60)<br>- ab5169, from the website (https://www.abcam.com/histone-h3-phospho-s28-antibody-ab5169.html): "This antibody is specific for Histone H3 phosphorylated at residue Ser 28 and does not recognise the unmodified residue or another phosphorylated residue (Ser 10) on the same histone."<br>- anti-PfGAP45 pulls down the predicted members of the Plasmodium falciparum glideosome complex in IPs (Jones, M. L., et al (2009); Molecular and Biochemical Parasitology. 168, 55–62)<br>- anti-PfRhopH3 mAb (No. 87) recognizes the expected band in the high molecular weight rhoptry complex (Doury, J. C., et al (2009) Parasitology. 108, 269–280)<br>- anti-MTIP labels the inner membrane complex by IFA and recognizes a single spot by 2D-SDS-PAGE (Green, J.L., et al (2008); The Journal of Biological Chemistry. 283 (45): 30980-30989)<br>-anti-MSP1.19 recognizes the 19 kDa C-terminal fragment of MSP1 by immunoblot, pulls down a complex of merozoite surface antigens (Jana S. McBride and Hans-G. Heidrich (1987); Molecular and Biochemical Parasitology. 23 : 71-84) (Mickael J. Blackman et al. (1990). The Journal of Experimental Medecine. 172 (1): 379_382)<br>-anti-RON4 recognizes a single 225 kDa protein from P. falciparum extracts, labels the neck of the rhoptries by immuno-electron-microscopy and gives a characteristic punctate staining in schizonts by IFA (Roger N. et al. (1988). Molecular and Biochemical Parasitology. 27 : 137-141)<br>- anti-SUB1 specifically recognizes pfsub1 gene products by western-blot and localizes to granules by immuno-electron microscopy (Michael J. Blackman et al. (1998). The Journal of Biological Chemistry. 273 (36): 23398–23409)<br>- anti-AMA1 antibodies specifically recognize AMA1 by immunoblot and pulls down the moving-junction complex comprising AMA1 associated with RONs proteins (Collins R.C. et al. (2009). PLoS Pathogens. 5 (1): e1000273<br>- ab38905 consistently recognizes a protein of 40 kDa, corresponding to P. falciparum aldolase, as reported also by others (see references on Abcam website)<br>- anti-GFP antibody 11814460001 from Roche is a "Mixture of two high-affinity monoclonal antibodies selected for their performance in detection of GFP and GFP fusion proteins." (https://www.sigmaaldrich.com/content/dam/sigma-aldrich/docs/Roche/Bulletin/1/11814460001bul.pdf)

Eukaryotic cell lines

Policy information about cell lines

Cell line source(s)	Plasmodium falciparum lines were obtained originally from WEHI Institute (Melbourne, Australia) or from Anthony Holder (Francis Crick Insitute, London, UK) as reported in the manuscript.
Authentication	All transgenic parasite lines used in this study were validated with PCR-based genotyping and sequencing, as reported in the manuscript.
Mycoplasma contamination	Parasite cell lines were not tested for mycoplasma contamination.
Commonly misidentified lines<br>(See ICLAC register)	none

Human research participants

Policy information about studies involving human research participants

Population characteristics	To culture P. falciparum parasites, we used human erythrocytes obtained as donations from anonymized individuals from the french Bloodbank (Etablissement Français du sang, France) or from a commercial source (Research Blood Components, Boston, USA).
Recruitment	We were not involved in the recruitment of the blood donors
Ethics oversight	French Bloodbank (Etablissement Français du sang, France) provides for the blood under the approval number 21PLER2016-0103; also Institutional Review Board (IRB) at Harvard*<br>* Because the human blood is de-identified and anonymous, IRB has determined that our research is NOT human subject research.

Note that full information on the approval of the study protocol must also be provided in the manuscript.

Flow Cytometry

Plots

Confirm that:

- [x] The axis labels state the marker and fluorochrome used (e.g. CD4-FITC).
- [x] The axis scales are clearly visible. Include numbers along axes only for bottom left plot of group (a 'group' is an analysis of identical markers).
- [x] All plots are contour plots with outliers or pseudocolor plots.
- [x] A numerical value for number of cells or percentage (with statistics) is provided.

Methodology

Sample preparation	For PfPP1-iKO parasites, cells were collected and fixed in 4% paraformaldehyde (PFA), before staining with SYBR-Green I. Following 2 washes in PBS, cells were further analysed by flow cytometry. PfPP1-DD and related control parasites were fixed in 4% PFA and 0.0075-0.015% glutaraldehyde before DNA staining with SYBR-Green I; for intraerythrocytic developmental analysis, parasites were permeabilized in Triton-X-100 and treated with RNase prior to DNA staining.
Instrument	For PfPP1-iKO parasites, FACS experiments were performed with a BD FACSCanto 1 cytometer. PfPP1-DD and related control parasites were measured with a MacsQuant 10 flow-cytometer (Miltenyi).
Software	For flow analysis, FlowJo, versions 10.5.3 and 10.6.2 were used.
Cell population abundance	Depending on the stage of the parasite development, infected red blood cells accounted for 0.2-17% of the population. We counted 20000-100000 cells for each sample, and the mean fluorescence intensity of the infected red blood cells were at least 10 times higher than for unifected red blood cells, giving confidence in the identification of the sorted population. Subpopulations of infected cells relevant to this study (i.e. rings and schizonts) are separated also from each other.
Gating strategy	Initial FSC/SSC gates were drawn near the single centroid of the population, so that roughly 70-90% of cells were included for further analysis. Single erythrocyte measurements are enriched by gating on SSC-W/SSC-H or FSC-H/FSC-A.<br>With SYBR-Green I staining, parasites carrying a single genome (lowest signal) are separated clearly from uninfected erythrocytes, with at least 10x higher fluorescence.

- [x] Tick this box to confirm that a figure exemplifying the gating strategy is provided in the Supplementary Information.
