# Supplementary material for: Co-option of Plasmodium falciparum PP1 for egress from host erythrocytes
Source: Nat Commun. 2020 Jul 15;11:3532. doi: 10.1038/s41467-020-17306-1 (PMC7363832; doi:10.1038/s41467-020-17306-1)
Supplement: Supplementary file 3 — Description of Additional Supplementary Files [file 41467_2020_17306_MOESM3_ESM.pdf]

Description of Additional Supplementary Files

File Name: Supplementary Data 1
Description: Proteomic analysis of PfPP1-DD parasites in late IDC-stage parasites. Columns in "Proteomics measurements" tab are explained in "Key" tab.

File Name: Supplementary Data 2
Description: A second proteomic analysis of PfPP1-DD parasites in late IDC-stage parasites. Columns in "Proteomics measurements" tab are explained in "Key" tab.

File Name: Supplementary Data 3
Description: Phosphoproteomic analysis of PfPP1-DD parasites in late IDC-stage parasites. Measurements are from same samples as in Supplementary Data 2. Columns in "Phosphoproteomics measurements" tab are explained in "Key" tab.
